# Prevalence of lower urinary tract symptoms, urinary incontinence and retention in Parkinson's disease: A systematic review and meta-analysis

**DOI:** 10.3389/fnagi.2022.977572

**Published:** 2022-09-12

**Authors:** Fang-Fei Li, Yu-Sha Cui, Rui Yan, Shuang-Shuang Cao, Tao Feng

**Affiliations:** ^1^Department of Neurology, Center for Movement Disorders, Beijing Tiantan Hospital, Capital Medical University, Beijing, China; ^2^China National Clinical Research Center for Neurological Diseases, Beijing, China; ^3^Department of Neurology, Beijing Chaoyang Hospital, Capital Medical University, Beijing, China

**Keywords:** Parkinson's disease, urinary incontinence, urinary retention, meta-analysis, review, prevalence, lower urinary tract symptoms (LUTS)

## Abstract

**Background:**

Lower urinary tract symptoms (LUTS) are common non-motor symptoms but are often overlooked in Parkinson's disease (PD). The prevalence of LUTS in PD is inconsistent among different studies.

**Objective:**

To estimate the prevalence of LUTS, urinary incontinence, and urinary retention in PD patients, then, investigate potential sources of inconsistency in prevalence estimation.

**Methods:**

We searched PubMed, EMBASE, and Web of Science databases from inception to May 2022. Studies reporting the prevalence of LUTS or LUTS subtypes in PD were included. Pooled prevalence of LUTS, LUTS subtypes, urinary incontinence, and urinary retention was calculated *via* random-effects models. Meta-regression and subgroup analyses were performed.

**Results:**

Of 7,358 studies after duplicate removal, a total of 73 studies comprising 14,937 PD patients were included. The pooled prevalence of LUTS was 61% (95% CI 53–69; 27 studies; *n* = 5,179), while the pooled prevalence of storage symptoms and voiding symptoms was 59% (44–73; 9 studies; *n* = 798) and 24% (14–33; 11 studies; *n* = 886), respectively. The pooled prevalence of urinary incontinence, retention and post-void residual (PVR) volume ≥ 100 ml were 30% (95% CI 22–39; 21 studies; *n* = 6,054), 27% (17–37; 14 studies; *n* = 1,991), and 4% (1–7; 5 studies; *n* = 439), respectively. The prevalence of LUTS, urinary incontinence, or urinary retention was significantly associated with diagnostic methods.

**Conclusion:**

LUTS and its subtypes present in a significant proportion of PD patients. It is necessary to use standardized and validated methods to detect and screen LUTS and its subtypes.

**Systematic review registration:** https://www.crd.york.ac.uk/prospero/display_record.php?ID=CRD42022311233, Identifier: CRD42022311233.

## Introduction

Parkinson's disease (PD) is the second most prevalent neurodegenerative disease characterized by both motor and non-motor symptoms. Lower urinary tract symptoms (LUTS) are common non-motor symptoms of PD. However, they are frequently neglected (Chaudhuri et al., 2006). LUTS are group of symptoms related to the lower urinary tract. Typical LUTS include storage symptoms (including urinary incontinence) and voiding symptoms (including urinary retention), which often emerge 5–6 years after the onset of motor symptoms in PD (Bonnet et al., 1997). The mechanism of LUTS may be related to PD neuropathology, especially the disruption of the dopamine D_1_-GABAergic direct and bypass pathway (Sakakibara et al., 2016). α-synuclein, the pathological hallmark of PD, has been found in the pontine, sacral spinal cord, pelvic plexus, and genitourinary tract of PD patients (Wakabayashi and Takahashi, 1997; Beach et al., 2010). The structures responsible for normal bladder control, including pre-ganglionic, post-ganglionic sympathetic neurons, sacral parasympathetic nuclei, and frontal cortex were even proven to have PD neuropathology (Oyanagi et al., 1990; Braak et al., 2007; Tkaczynska et al., 2017).

LUTS exert immense impact on patients' life. The quality of life was physically and psychologically limited for PD patients with LUTS. The main effects were decline in self-esteem and social communication, in addition to depression, anxiety, deterioration of sexual life, and a decrease in physical activity (Farage et al., 2008). PD patients with LUTS showed elevated all-cause mortality and were more likely to develop severe complications including falls, disabling motor symptoms, cognitive dysfunction, and other non-motor dysfunction (Vaughan et al., 2013; Rana et al., 2015; Zhang and Zhang, 2015; Sakushima et al., 2016; Lee et al., 2018). Moreover, LUTS were correlated with increasing health-related costs. These can lead to serious burdens for PD patients, caregivers, and society (Mohammed and Ragab, 2010). This urges the need for an up-to-date estimation of the prevalence of LUTS. Establishing the prevalence of LUTS may not only raise awareness of early interventions but also help refine diagnostic criteria and differential diagnosis of PD and other parkinsonian syndromes. This may also guide effective planning of medical services.

However, there was considerable heterogeneity in the prevalence of LUTS in PD, ranging from 27 to 85% as shown in previous reports (Winge et al., 2006; Sakakibara et al., 2008; Jain, 2011; Martinez-Ramirez et al., 2020). Previous studies reporting the prevalence of LUTS were predominantly cross-sectional, with limited focus on the nature of LUTS. Furthermore, methods of determining the presence of LUTS are diverse, potentially leading to different reported rates. Urinary incontinence and retention are crucial symptoms of LUTS. PD patients were generally considered less likely to develop those two symptoms, and the presence of unexplained voiding difficulties with elevated post-void residual (PVR) volume ≥ 100 ml or unexplained urinary urge incontinence was often considered to support the diagnosis of multiple system atrophy (MSA) (Yamamoto et al., 2016; Wenning et al., 2022). However, urinary incontinence and retention are not rare, even PVR volume ≥ 100 ml can also occur in PD (Irene, 2019; Tateno et al., 2021). For example, one study with large sample size found that the prevalence of urinary incontinence was 43% in PD (Wüllner et al., 2007). Utilizing the Danish Prostate Symptom Score (Dan-PSS), the prevalence of urge incontinence in PD was up to 65.8% (Akkoç et al., 2017). Another study found the prevalence of incomplete bladder emptying was 75.5% in PD (Irene, 2019). Until now, no meta-analysis has been carried out to estimate the overall prevalence of LUTS, urinary incontinence, and retention in PD.

We aimed to conduct a systematic review and meta-analysis to determine the prevalence of the overall LUTS and its subtypes including urinary incontinence and retention in PD patients, as well as to explore potential sources of heterogeneity across prevalence estimates.

## Materials and methods

This systematic review and meta-analysis followed the Preferred Reporting Items for Systematic Reviews and Meta-Analysis (PRISMA) guidelines (Barendregt et al., 2013) (Supplementary Table 1). The protocol has been registered on PROSPERP (registration number: CRD42022311233).

### Date source and strategy

We screened PubMed, EMBASE, and Web of Science databases to identify relevant research from the outset until May 2022 using the following MeSH (Medical Subject Heading) terms and keyword variations: (“urination disorders” OR “urinary bladder, neurogenic” OR “urinary retention” OR “overactive bladder symptom” OR “nocturia” OR “urinary bladder, overactive” OR “urinary incontinence” OR “lower urinary tract symptoms” OR “urinary dysfunction” OR “dysuria”) AND (“Parkinson's disease” OR “Parkinsonism” OR “Parkinsonian” OR “Parkinson's disease”). Afterwards, endnote was utilized to integrate the citations from each database and additional eligible publications.

### Eligibility criteria and study selection

Studies were eligible if they met the following criteria: (1) published in peer-reviewed English journals; (2) participants were diagnosed according to UK Parkinson's Disease Society Brain Bank Diagnostic Criteria (Hughes et al., 1992) or MDS clinical diagnostic criteria for Parkinson's disease (Postuma et al., 2015); (3) reporting the prevalence of LUTS or LUTS subtypes; (4) LUTS or LUTS subtypes assessed by validated scales administered by experienced clinicians, self-report questionnaire, or published criteria from classification codes/definition; (5) prospective cohort study or cross-sectional study.

The studies were excluded if they: (1) did not provide full text; (2) included insufficient or unclear fragmented data for analysis; (3) enrolled patients who have been diagnosed with prostate carcinoma, uncontrolled diabetes, as well as any other diseases that cause urinary problems, or taken drugs such as diuretics; (4) with small sample size (*n* < 20). (5) duplicated publications; (6) systematic reviews, meta-analyses, letters, protocols. When results on the same dataset were reported in several publications, only the most complete publication was included in the analysis. Two independent observers (FFL, YSC) evaluated the results and resolved any disagreement by discussion or with recourse to a third arbitrator (TF).

### Quality assessment

Two authors (FFL, YSC) independently assessed study quality and risk of bias using the Newcastle-Ottawa Scale (NOS) (Stang, 2010) for cohort study and the Agency for Healthcare Research and Quality (AHRQ) (Williams et al., 2010) for cross-sectional study in Table 1. The highest score was 9 points for the cohort and case-control studies: low quality = 0–4 points; moderate quality = 5–7 points; high quality = 8–9 points. The highest score was 11 points for cross-sectional studies: low quality = 0–3 points; moderate quality = 4–7 points; high quality = 8–11 points. Higher score indicates better quality.

**Table 1:** Characteristics of studies included in the meta.

**First author and year**	**Country**	**Region**	**Study design**	**Study site**	**LUTS diagnosis standard**	**Sample size**	**Male/female**	**Mean age (years)**	**Disease duration (years)**	**Quality assessment**
Hattori (1992)	Japan	The other	Cross-sectional	Single-center	Definition	110	43/67	58.8	4.3	Low
Singe (1992)	USA	North American	Cross-sectional	Single-center	Questionnaire	48	48/0	65.9 (1.46)	7.76 (0.91)	Low
Araki (2000)	Japan	The other	Cross-sectional	Single-center	IPSS	208	82/121	66.6	9.2	Moderate
Sakakibara (2001)	Japan	The other	Cross-sectional	Single-center	Questionnaire	115	52/63	59	6	Low
Campos (2003)	Brazil	The other	Cross-sectional	Single-center	AUA-SI	61	31/30	59.6	4.9	Low
Hobson (2003)	UK	European	Cross-sectional	Community-based	Questionnaire	123	78/45	75.1 (9.3)	7.8 (8.01)	Low
Hahn (2005)	Germany	European	Cross-sectional	Single-center	Questionnaire	20	8/12	64.3 (6.7)	6.3 (4.2)	Low
Winge (2006)	Denmark	European	Cohort	Single-center	Dan-PSS/IPSS	107	64/43	62.8	8	High
Wüllner (2007)	Germany	European	Cross-sectional	Multicenter	Questionnaire	3,414	2,076/1,338	66.07	9.02 (6.36)	Moderate
Verbaan (2007)	Netherlands	European	Cohort	Single-center	SCOPA-AUT	420	269/151	61.6 (11.5)	10.5 (6.5)	Moderate
Cheon (2008)	Korea	The Other	Cross-sectional	Single-center	NMSS	74	28/46	64.9 (8.6)	6.4 (6.1)	Low
Coelho (2010)	Spain	European	Cross-sectional	Multicenter	Questionnaire	50	23/27	74.1 (7.0)	17.94 (6.3)	Moderate
Mohammed (2010)	Egypt	The other	Cross-sectional	Single-center	IPSS	49	31/18	63.73 (7.21)	7.81 (3.27)	Low
Swaminath (2010)	India	The other	Cross-sectional	Single-center	Questionnaire	150	111/39	57.4 (12)	1.9 (1.63)	Low
Muller (2011)	Norway	European	Cohort	Single-center	Questionnaire	207	122/85	67.9 (42.3–88.1)	2.3 (1.8)	Low
Ragab (2011)	Ghana	The other	Cross-sectional	Single-center	IPSS	49	31/18	63.73 (7.21)	7.81 (3.27)	Low
Soliman (2011)	Egypt	The other	Cross-sectional	Single-center	IPSS	25	43/67	60.8 (8.3)	4.5 (2.7)	Moderate
Uchiyama (2011)	Japan	The other	Cross-sectional	Single-center	questionnaire	30	30/0	66.7 (8.4)	1.97 (1.93)	Low
Yamamoto (2011)	Japan	The other	Cross-sectional	Single-center	questionnaire	61	38/23	67	3.2	Low
Crosiers (2012)	Belgium	European	Cross-sectional	Community-based	NMSS	215	83/132	67.1 (10.4)	7.3 (6.3)	Low
Bostantjopoulou (2013)	Greece	European	Cross-sectional	Single-center	NMSS/questionnaire	166	109/55	59.5 (9.3)	7.09 (5.31)	Moderate
Guo (2013)	China	The other	Cross-sectional	Single-center	NMSS	616	347/269	61.54 (10.98)	4.76 (4.18)	Low
Khoo (2013)	UK	European	Cohort	Single-center	NMSS	159	347/269	66.6 (10.3)	0.36	Moderate
Špica (2013)	Serbia	The other	Cross-sectional	Single-center	NMSS	208	140/58	60.7 (11.4)	9.04 (6.02)	Low
Vaughan (2013)	USA	North American	Cross-sectional	Single-center	IPSS/definition	60	39/21	63.4 (8.99)	–	Moderate
Weerkamp (2013)	Netherlands	European	Cross-sectional	Multicenter	NMSS	73	33/40	78.8	10.1	Moderate
Zhou (2013)	China	The other	Cross-sectional	Single-center	NMSS	230	136/94	67.7	4.7	Moderate
Tsujimura (2014)	Japan	The other	Cross-sectional	Single-center	OABSS	161	161/0	71.4 (8.2)	8.9 (5.1)	Low
Vongvaivanich (2014)	Thailand	The other	Cross-sectional	Single-center	NMSS	165	60/55	68.77 (11.59)	4.64 (3.85)	Low
de Souza (2015)	India	The other	Cross-sectional	Single-center	definition	171	102/69	67.1	4.69	Moderate
M. Liu (2015)	Taiwan	The other	Cohort	Single-center	NMSS	210	101/70	66.1 (9.86)	6.11 (4.13)	Low
Z.Liu (2015)	China	The other	Cross-sectional	Single-center	Questionnaire	58	35/23	66.8 (48–80)	5.4 (1–12)	Low
Raven (2015)	India	The other	Cross-sectional	Single-center	NMSS	81	50/31	62.93 (10.93)	–	Moderate
Rana (2015)	Canada	European	Cross-sectional	Community-based	Definition	314	177/137	75 (10.58)	–	Moderate
Telarovic (2015)	Croatia	European	Cross-sectional	Single-center	Questionnaire	110	54/56	58 (11.49)	6 (4.72)	Low
Vale (2015)	Brazil	The other	Cross-sectional	Single-center	Questionnaire	30	17/13	67.3 (7.5)	–	High
M. Zhang (2015)	China	The other	Cross-sectional	Single-center	Definition	91	17/13	68.3	12.2	Low
Zis (2015)	UK	European	Cross-sectional	Multicenter	NMSS	234	149/85	67.74 (11.23)	3.21 (1.43)	Low
Benli (2016)	Turkey	European	Cross-sectional	Single-center	IPSS/OABSS	39	20/19	69.7 (7.4)	5.36 (3.5)	Low
Mekawichai (2016)	USA	North American	Cross-sectional	Single-center	NMSS	136	73/63	63.1 (10.2)	4.99	Moderate
Mito (2016)	Japan	The other	Cross-sectional	Single-center	OABSS	31	12/19	72 (6.7)	1.9 (1.8)	Low
Ou (2016)	China	The other	Cohort	Single-center	NMSS	117	68/49	60.1 (11.8)	3.9 (3.6)	High
Sakushima (2016)	Japan	The other	Cohort	Single-center	OABSS	97	40/57	71.5 (7.3)	7.63 (5.3)	Moderate
Smith (2016)	UK	European	Cross-sectional	Single-center	Questionnaire	23	17/6	68.5 (50–85)	10.1	Low
Yamamoto (2016)	Japan	The other	Cross-sectional	Single-center	Questionnaire	218	134/84	66.2 (0.46)	3.2	Low
S. Zhang (2016)	China	The other	Cross-sectional	Single-center	NMSS	454	260/194	61.54 (10.98)	4.76 (4.18)	Moderate
Akkoç (2017)	Turkey	European	Cross-sectional	Multicenter	Dan-PSS	73	49/24	68 (35–87)	4 (1–19)	Moderate
Tkaczynska (2017)	Germany	European	Cross-sectional	Single-center	Questionnaire	94	60/34	71 (56–89)	6.4 (1.2–24.4)	Low
Radicati (2017)	Italy	European	Cross-sectional	Single-center	NMSS	100	60/40	69.19 (8.27)	3.83 (2.25)	Low
Yamamoto (2017)	Japan	The other	Cross-sectional	Single-center	Questionnaire	91	41/50	67.03 (0.76)	7.16 (0.54)	Low
Kim (2018)	Korea	The other	Cross-sectional	Single-center	IPSS	79	36/43	72.4 (8.0)	6.8 (4.4)	Moderate
Lee (2018)	Korea	The other	Cross-sectional	Single-center	SCOPA-AUT	163	93/70	68.9 (9.9)	1.0 (1.6)	Low
Mito (2018)	Japan	The other	Cross-sectional	Single-center	OABSS	31	12/19	71.2 (6.7)	2.4 (2.6)	Low
Mukhtar (2018)	Pakistan	The other	Cross-sectional	Single-center	NMSS	85	70/15	57.61 (10.64)	–	Moderate
Serra (2018)	Europe and Australia	Europe and Australia	Cross-sectional	Multicenter	SCOPA-AUT	423	275/148	61 (9.7)	–	Low
Valentino (2018)	Italy	European	Cross-sectional	Single-center	SCOPA-AUT	48	28/20	62.7 (10.6)	6.2 (4.2)	Moderate
Valldeoriola (2018)	Spain	European	Cohort	Multicenter	NMSS	378	215/163	70.2 (9.9)	6.1 (4.8)	Moderate
Aldaz (2019)	France	European	Cross-sectional	Single-center	NMSS	45	22/23	66.13 (9.95)	10.11 (6.7)	Low
Fanciulli (2019)	Italy	European	Cross-sectional	Single-center	Questionnaire	70	–	–	–	Low
Irene (2019)	Romania	European	Cross-sectional	Multicenter	SCOPA-AUT	86	48/38	70.6	6.33	Low
Sanchez (2019)	Spain	European	Cross-sectional	Single-center	NMSS	120	88/32	63.33 (8.6)	8 (5–13)	Moderate
Shin (2019)	Korea	The other	Cross-sectional	Single-center	Questionnaire	112	55/57	68	7.6 (4.6)	Moderate
Stanković (2019)	Serbia	The other	Cross-sectional	Single-center	SCOPA-AUT	107	59/48	61.5 (9.6)	2.2	Moderate
Xu (2019)	China	The other	Cross-sectional	Single-center	OABSS	100	55/45	65.97 (8.247)	4 (2.7)	Moderate
Zong (2019)	China	The other	Cross-sectional	Single-center	AUA-SI/OABSS	416	307/109	61.2 (5.9)	7.7 (4.1)	Moderate
Martinez (2020)	Mexico	North American	Cross-sectional	Multicenter	SCOPA-AUT	414	274/140	61.1 (9.7)	0.56 (0.55)	Moderate
Nakahara (2020)	Japan	The other	Cross-sectional	Single-center	Definition	91	34/57	75 (67–80)	9 (5–13)	Low
Schrag (2020)	Europe	European	Cross-sectional	Multicenter	NMSS	692	373/319	76.1 (8.4)	15.4 (7.7)	Moderate
Tkaczynska (2020)	Germany	European	Cross-sectional	Single-center	Questionnaire	189	93/96	64.7 (7.9)	5.1 (3.8)	Low
Ayele (2021)	Ethiopia	The other	Cross-sectional	Multicenter	NMSS	123	89/34	62.9 (10.4)	4 (2–6)	Low
Lichter (2021)	USA	North American	Cross-sectional	Multicenter	NMSS	164	112/52	72.05 (9.91)	8.9 (5.98)	Moderate
Ojo (2021)	Nigeria	The other	Cross-sectional	Multicenter	NMSS	825	604/221	63.7 (10.1)	3	High
Tateno et al. (2021)	Japan	The other	Cross-sectional	Single-center	OABSS/IPSS/questionnaires	30	18/12	69.5	1.29	Low

IPSS, the International Prostate Symptom Score; AUA-SI, the American Urological Association Symptom Index; OABSS, the overactive bladder symptom score; NMSS, the Non-Motor Symptom Scale; SCOPA-AUT, Scale for Outcomes in Parkinson's Disease for Autonomic Symptoms; DAN-PSS, the Danish Prostate Symptom Score.

### Data extraction

Data extraction was performed independently by two authors (FFL, YSC), using a standardized data collection spreadsheet in EXCEL. The following items were extracted: study characteristics (first author, publication year, country/region, study design, source), participant characteristics (age, disease duration, sample size, number of patients with LUTS or LUTS subtypes, gender of participants and H&Y stage), and methods used to evaluate LUTS or its subtypes (definitions, urinary dysfunction questionnaires or clinical scales).

LUTS subtypes include urinary storage symptoms [urgency, Overactive bladder (OAB) syndrome, pollakiuria, frequency, nocturia, incontinence], urinary voiding symptoms (dysuria, hesitancy, prolongation, intermittency, weak stream, retention, straining to void), and post-voiding symptoms according to the International Continence Society (ICS) report (D'Ancona et al., 2019). The clinical scales contain the overactive bladder symptom score (OAB-SS) (Homma et al., 2006), the International Prostate Symptom Score (IPSS) (Araki and Kuno, 2000), the Non-Motor Symptom Scale (NMSS) (Koh et al., 2012), Scale for Outcomes in Parkinson's Disease for Autonomic Symptoms (SCOPA-AUT) (Kim et al., 2017), the Danish Prostate Symptom Score (Dan-PSS) (Akkoç et al., 2017), and the American Urological Association Symptom Index (AUA-SI) (Barry et al., 1992). If disagreements could not be resolved through careful discussion by two investigators (FFL, YSC), a consensus was achieved by the involvement of a third investigator (TF) when necessary.

### Statistical analysis

The meta-analysis was performed with Rx64 4.1.0 and RStudio using the “Matrix”, “Meta,” and “metaphor” packages to analyze pooled prevalence of LUTS or its subtypes with 95% confidence intervals (CIs). Heterogeneity for the pooled estimate of prevalence were quantified using the *I*^2^ statistic, and its significance was measured using the Q test *p*-value. The random-effects model was adopted if significant heterogeneity were detected (*I*^2^ > 50% or *p* > 0.1). Otherwise, the fixed-effects model was used. Meta-regression was performed to assess the effects of following variables: age, disease duration, regions of the participant, quality of studies and diagnostic method. We conducted subgroup analyses using diagnosis criteria (Scales, questionnaire, and definition), gender (proportion of female), H&Y stage (<3 and ≥3), study site (single-center, multicenter, and community-based), study design (cross-sectional and cohort), mean age (<65 and ≥65) of participants, region (North American, European, and the other) and quality of studies (Low, moderate and high). Sensitivity analyses were performed to evaluated the robust of the synthesis results by leave-one-out method. Publication bias was checked by funnel plot and Egger's test. The trim-and-fill computation was used to estimate the effect of publication bias on the interpretation of the results. Statistical significance was set at two-tailed *p* < 0.05.

## Results

### Characteristics of the included studies

**Figure 1:**
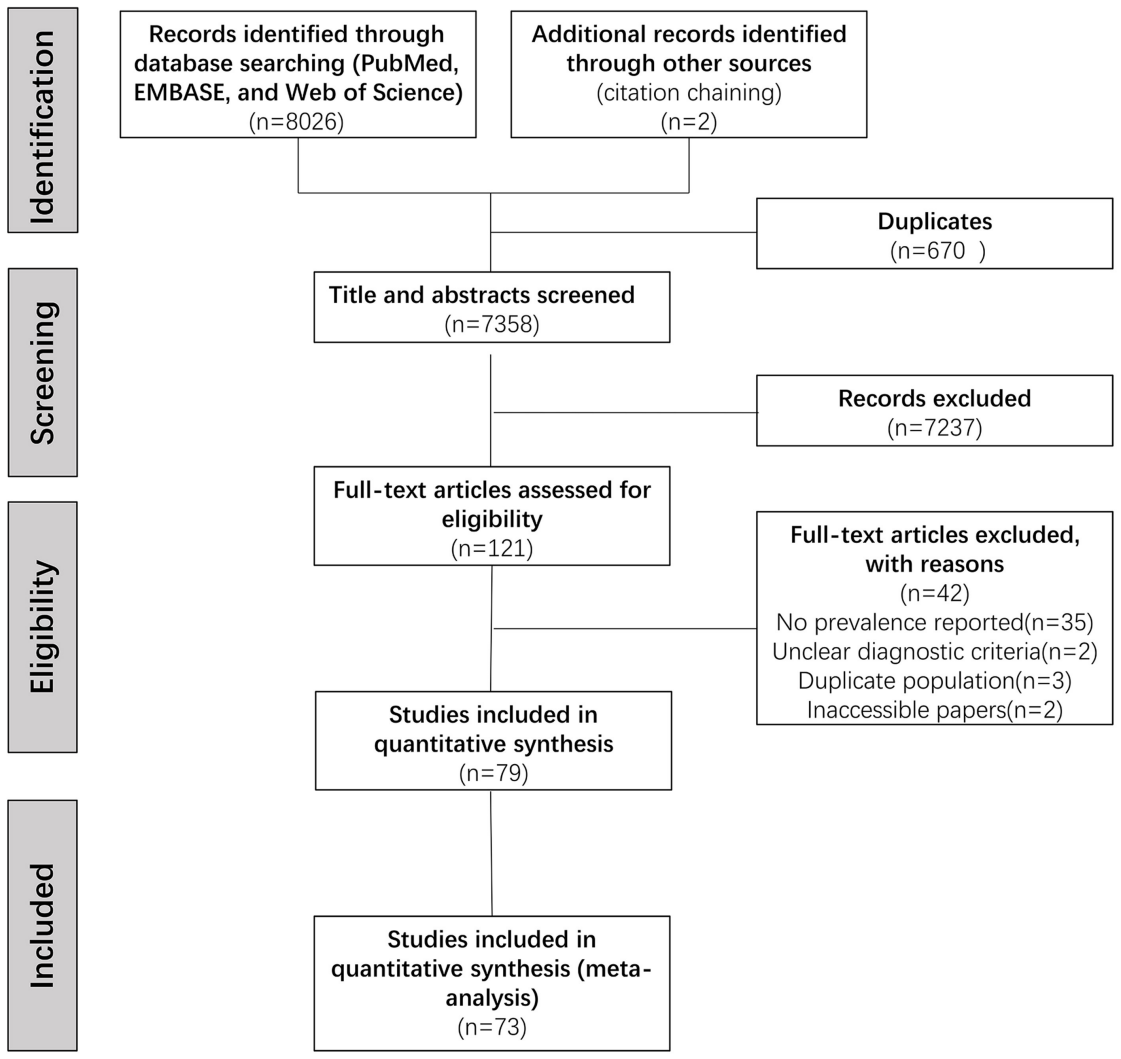
Study selection flowchart, performed according to the PRISMA 2020 guidelines.

After duplicating removal, we identified 7,358 articles through the database searching. Screening titles and abstracts led to the elimination of 7,237 irrelevant articles, full-text versions of the remaining 121 potentially eligible articles were assessed. Of those, 79 articles were included in the qualitative synthesis. Overall, 73 studies comprising 14,937 PD patients were identified eligible for the meta-analysis (Supplementary Table 2). The procedure was shown flow chart in Figure 1.

Sample size ranged from 20 to 3,414. Average age of participants ranged from 57.4 to 76.1 years. The average disease duration of PD ranged from 0.36 to 17.94 years. Forty-seven studies used the scales, 19 used questionnaire, 4 used definitions, and 3 applied mixed methods. We classified 4 studies as high quality, 29 studies as moderate, and 40 studies as low. The results indicated that the overall quality of the included articles was relatively low. There were 65 cross-sectional studies and 8 cohort studies in the included research. The studies were conducted in 29 countries, 27 performed in Europe, 5 in North American, while others were undertaken in the other regions (*n* = 40). Table 1 shows the characteristics of included study.

### Prevalence of LUTS in PD

**Figure 2:**
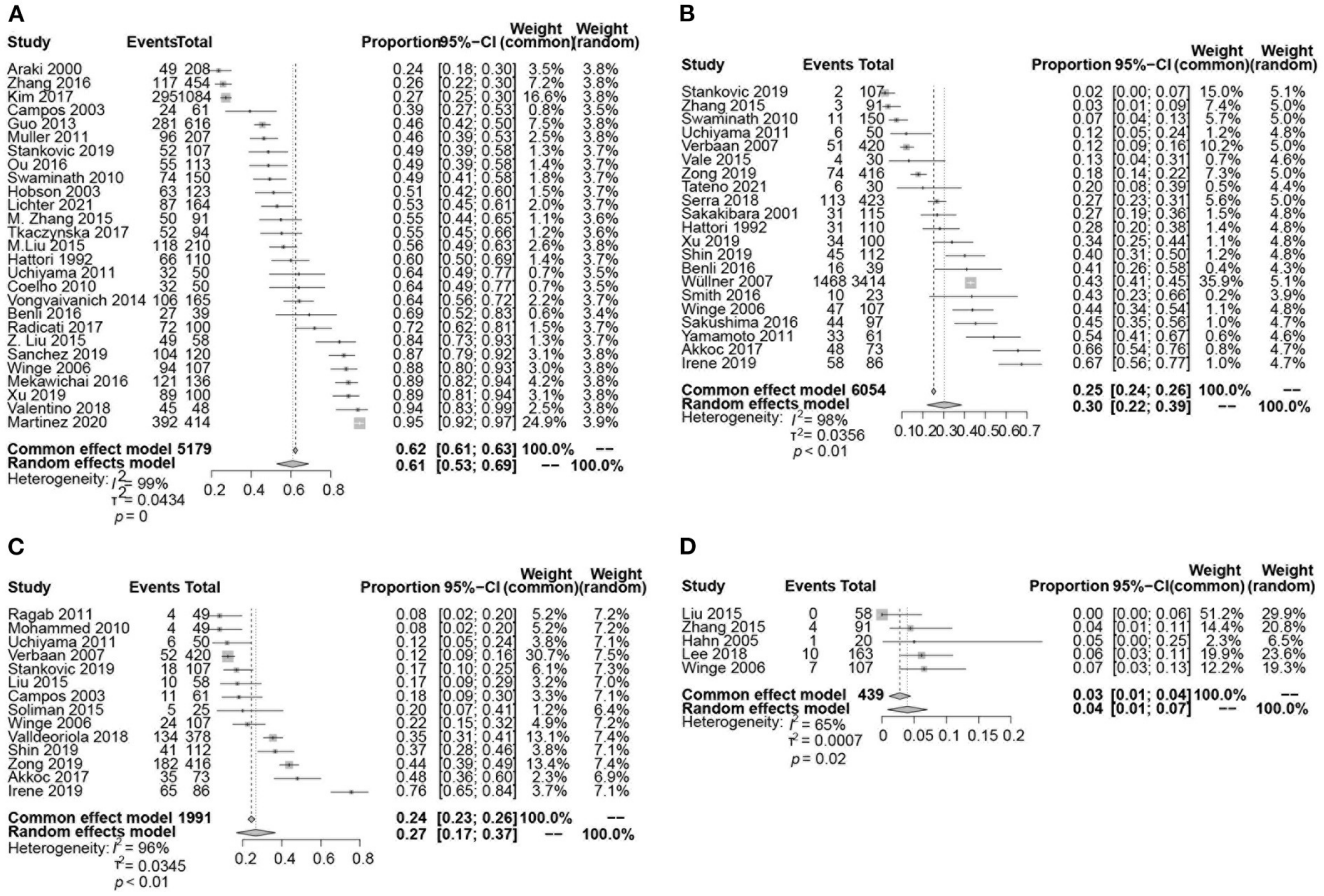
Forest plot showing the prevalence of LUTS **(A)**, urinary incontinence **(B)**, urinary retention **(C)**, and post-void residual (PVR) volume ≥ 100 ml **(D)** in PD patients.

**Figure 3:**
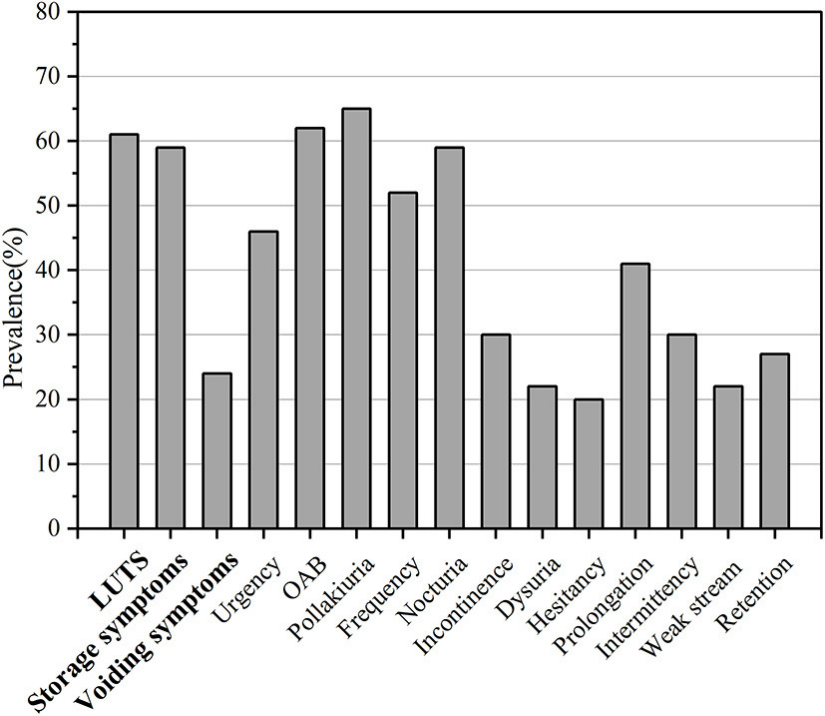
Frequency of LUTS or its subtypes in PD patients. The x-axis shows different kinds of LUTS while the y-axis shows the percentage.

The pooled prevalence of LUTS was 61% (95% CI 53–69; *I*^2^ = 99%; 27 studies; *n* = 5,179; Figures 2A, 3).

For subgroup analyses, we found that H&Y stage, gender, and different diagnostic tools may be cause of heterogeneity related to the prevalence of LUTS (Table 2). The pooled prevalence of LUTS was 59% (95% CI 48–71; *I*^2^ = 68%; 3 studies; *n* = 277) in PD with H&Y stage <3, whereas 70% (95% CI 52–88; *I*^2^ = 92%; 4 studies; *n* = 194) in PD with H&Y stage ≥ 3. The pooled prevalence of LUTS was 62% (95% CI 47–77; *I*^2^ = 96%; 6 studies; *n* = 839) in male PD patients, whereas 54% (95% CI 41–67; *I*^2^ = 78%; 5 studies; *n* = 352) in female PD patients.

**Table 2:** Prevalence of LUTS or subtypes: results of the subgroup analysis.

**Subgroup prevalence, % (95% CI)**, ***n*** **studies**, ***N*** **number**, ***I**^**2**^*												
	**LUTS**				**Incontinence**				**Retention**			
	**Prevalence, % (95% CI)**	***n***	***N***	***I**^2^* **(%)**	**Prevalence, % (95% CI)**	***n***	***N***	***I**^2^* **(%)**	**Prevalence, % (95% CI)**	***n***	***N***	***I**^2^* **(%)**
**Region**												
North American	79 (54–100)	3	714	98	–	–	–	–	–	–	–	–
European	68 (57–79)	10	995	95	32 (18–46)	9	4,649	99	35 (16–54)	6	1,171	98
The other	52 (42–63)	14	3,470	98	29 (18–40)	12	1,405	96	21 (11–30)	8	820	94
	*p* = 0.05				*p* = 0.75				*p* = 0.19			
**Methods**												
Scales	62 (51–72)	19	4,340	99	34 (21–46)	11	1,898	98	28 (16–40)	11	1,771	97
Questionnaire	60 (48–71)	6	638	90	28 (14–42)	7	3,848	98	22 (7–37)	1	50	–
Definition	58 (51–65)	2	201	0	28 (20–38)	1	110	–	–	–	–	–
Urodynamic tests	–	–	–	–	21 (0–58)	2	203	98	27 (8–46)	2	170	88
	*p* = 0.84				*p* = 0.86				*p* = 0.08			
**Disease severity**												
H&Y stage <3	59 (48–71)	3	277	68	20 (15–25)	3	256	0	16 (3–29)	2	240	76
H&Y stage ≥ 3	70 (52–88)	4	194	92	48 (24–71)	3	266	84	41 (7–75)	2	256	97
	*p* = 0.33				***p*** **=** **0.03**				*p* = 0.19			
**Age(mean)**												
<65	64 (50–78)	12	2,436	99	20 (11–29)	8	1,848	96	19 (10–27)	8	1,234	95
≥65	58 (49–67)	15	2,743	98	37 (26–48)	13	4,206	98	37 (19–56)	6	757	96
	*p* = 0.47				***p*** **=** **0.02**				*p* = 0.07			
**Gender**												
Male	62 (47–77)	6	839	96	31 (20–41)	7	2,579	87	21 (1–41)	4	414	95
Female	54 (41–67)	5	352	78	43 (25–62)	6	1,555	94	25 (3–47)	3	87	81
	*p* = 0.44				*p* = 0.25				*p* = 0.78			
**Study site**												
Single-center	60 (51–68)	24	4,592	98	29 (20–37)	19	5,545	98	22 (14–30)	12	1,527	94
Multicenter	80 (50–100)	2	464	95	47 (7–87)	2	509	98	55 (16–95)	2	464	98
Community-based	51 (42–60)	1	123	–	–	–	–	–	–	–	–	–
	*p* = 0.12				*p* = 0.38				*p* = 0.10			
**Quality assessment**												
High-moderate	58 (43–73)	12	3,175	100	27 (10–43)	7	1,243	97	28 (18–39)	7	1,526	96
Low	63 (55–71)	15	2,022	95	32 (23–42)	14	4,811	98	25 (7–43)	7	465	97
	*p* = 0.55				*p* = 0.58				*p* = 0.76			

Statistically significant values are reported in bold.

Different diagnostic tools also influenced prevalence of LUTS. The pooled prevalence of LUTS based on definitions was 58% (95% CI 51–65; 2 studies; *n* = 201) with considerable heterogeneity (*I*^2^ = 0%; *p* = 0.47). Using NMSS, the pooled prevalence of LUTS was 60% (95% CI 48–72; 10 studies; *n* = 2,172), also with considerable heterogeneity (*I*^2^ = 98%; *p* < 0.01). Using IPSS, the pooled prevalence of LUTS was 39% (95% CI 11–67) in 1,331 PD patients reported by 3 studies, with considerable heterogeneity (*I*^2^ = 94%; *p* < 0.01). Using SCOPA-AUT, the pooled prevalence of LUTS was 79% (95% CI 50– 100) estimated from 569 PD patients, with considerable heterogeneity of these 3 studies (*I*^2^ = 98%; *p* < 0.01). Using other questionnaires of LUTS diagnostic tools, the pooled prevalence of LUTS was 60% (95% CI 48–71) of 638 PD reported by 6 studies, with considerable heterogeneity (*I*^2^ = 90%; *p* < 0.01). The prevalence of LUTS was 89% using OABSS (*n* = 100), 39% using AUA-SI (*n* = 61), and 88% using Dan-PSS (*n* = 107), respectively, all from one study (Supplementary Figure S1; Supplementary Table 3).

By meta-regression analyses, region (*p* = 0.016) and number of PD patients (*p* = 0.031) were causes of heterogeneity related to the prevalence of LUTS, while the study site, quality assessment, age, and disease duration were not.

No significant publication bias was found by Begg's funnel plot (*p* = 0.695).

### Prevalence of LUTS subtypes in PD

#### Storage symptoms

The pooled prevalence of storage symptoms was 59% (95% CI 44–73; 9 studies; 798 PD; *I*^2^ = 98%; *p* < 0.01; Table 3; Figure 3; Supplementary Figure S2).

**Table 3:** Prevalence of LUTS subtypes in PD.

	***n* studies**	***N* number**	**Range**	**Prevalence (%), 95% CI**	***I*^2^ (*p*)**	**Egger's funnel plot for Publication bias**	**Trim and Fill**
Storage symptoms	9	798	32–95	59 (44–73)	98 (<0.01)	–	–
Urgency	42	7,292	11–77	46 (41–51)	97 (<0.01)	*p* = 0.097	0
OAB	7	388	35–100	62 (44–80)	96 (<0.01)	–	–
Pollakiuria	2	177	47–83	65 (30–100)	96 (<0.01)	–	–
Frequency	27	4,591	21–87	52 (44–60)	97 (<0.01)	*p* = 0.076	0
Daytime-frequency	12	1,044	5–80	41 (29–53)	96 (<0.01)	*p* < 0.001	6
Nighttime-frequency	9	806	19–87	53 (37–70)	98 (<0.01)	–	–
Nocturia	40	7,784	21–95	59 (54–65)	97 (<0.01)	*p* = 0.835	0
Incontinence	21	6,054	2–67	30 (22–39)	98 (<0.01)	*p* = 0.861	0
Urge incontinence	13	1,422	7–66	32 (23–41)	94 (<0.01)	*p* = 0.007	6
Voiding symptoms	11	886	7–59	24 (14–33)	93 (<0.01)	*p* = 0.027	5
Dysuria	4	671	11–36	22 (11–34)	94 (<0.01)	–	–
Hesitancy	6	724	2–40	20 (7–32)	96 (<0.01)	–	–
Prolongation	3	184	12–72	41 (7–75)	97 (<0.01)	–	–
Intermittency	8	563	6–87	30 (9–51)	97 (<0.01)	–	–
Weak stream	8	494	2–83	22 (4–39)	95 (<0.01)	–	–
Retention	14	1,991	8–76	27 (17–37)	96 (<0.01)	*p* = 0.492	0
PVR > 100 ml	5	439	0–7	4 (1–7)	65 (0.02)	–	–

OAB, overactive bladder; PVR, post-void residual.

##### Incontinence

The pooled prevalence of urinary incontinence was 30% (95% CI 22–39; *I*^2^ = 98%; *p* < 0.01; 21 studies; *n* = 6,054; Figure 2B). The pooled prevalence of urge incontinence was 32% (95% CI 23–41; *I*^2^ = 94%; *p* < 0.01; 13 studies; *n* = 1,422).

Using urodynamic tests, the pooled prevalence of urinary incontinence was 21% (95% CI 0–58; 2 studies; *n* = 203), with significant heterogeneity (*I*^2^ = 98%; *p* < 0.01). Using clinical scales, the pooled prevalence of urinary incontinence was 34% (95% CI 21–46; 11 studies; *n* = 1,898), with significant heterogeneity (*I*^2^ = 98%; *p* < 0.01); The pooled prevalence of urinary incontinence was 28% (95% CI 14–42; *I*^2^ = 98%; *p* < 0.01; 7 studies; *n* = 3,843) using questionnaires and 28% using definition in one study.

The pooled prevalence of urinary incontinence was 20% (95% CI 15–25; *I*^2^ = 0%; 3 studies; *n* = 256) in PD with H&Y stage <3, whereas 48% (95% CI 24–71; *I*^2^ = 84%; 3 studies; *n* = 266) in PD with H&Y stage ≥ 3. The pooled prevalence of urinary incontinence was 20% (95% CI 11–29; *I*^2^ = 96%; 8 studies; *n* = 1,848) in PD with age <65 years, whereas 37% (95% CI 26–48; *I*^2^ = 98%; 13 studies; *n* = 4,206) in PD with age ≥ 65 years (Table 2).

By meta-regression analyses, age (*p* = 0.017) was a source of heterogeneity related to the prevalence of urinary incontinence, while the study site, quality assessment, region, number of PD patients, and disease duration were not.

**OAB** The pooled prevalence of OAB was 62% (95% CI 44–80; *I*^2^ = 96%; *p* < 0.01; 7 studies; *n* = 388) (Supplementary Figure S3).

##### Urinary urgency

The pooled prevalence of urgency was 46% (95% CI 41–51; *I*^2^ = 97%; *p* < 0.01; 46 studies; *n* = 7,292) (Supplementary Figure S4).

By subgroup analysis, we found that H&Y stage, sex, and different diagnostic tools may be factors affecting the prevalence of urgency. By meta-regression analyses, region (*p* = 0.046) was a source of heterogeneity, while the study site, quality assessment, age, number of PD patients, and disease duration were not.

##### Urinary frequency

The pooled prevalence of frequency was 52% (95% CI 44–60; *I*^2^ = 97%; *p* < 0.01; 27 studies; *n* = 4,591). The pooled prevalence of daytime frequency was 41% (95% CI 29–53; *I*^2^ = 96%; *p* < 0.01; 12 studies; *n* = 1,044). The pooled prevalence of nighttime frequency was 53% (95% CI 37–70; *I*^2^ = 98%; *p* < 0.01; 9 studies; *n* = 806) (Supplementary Figure S5).

By meta-regression analyses, age (*p* = 0.028) was a cause of heterogeneity related to the prevalence of frequency, while study site, quality assessment, region, number of PD patients, and disease duration were not.

##### Nocturia

The pooled prevalence of nocturia was 59% (95% CI 54–65; *I*^2^ = 97%; *p* < 0.01; 40 studies; *n* = 7,784) (Supplementary Figure S6).

By subgroup analysis, we found that H&Y stage and different diagnostic tools may be factors affecting the prevalence of nocturia.

By meta-regression analyses, age (*p* = 0.026) was a cause of heterogeneity related to the prevalence of nocturia, while the study site, quality assessment, region, number of PD patients, and disease duration were not.

##### Pollakiuria

The pooled prevalence of pollakiuria was 65% (95% CI 30–100; *I*^2^ = 96%; *p* < 0.01; 2 studies; *n* = 177) (Supplementary Figure S7).

#### Voiding symptoms

The pooled prevalence of voiding symptoms was 24% (95% CI 14–33; 11 studies; *n* = 886; Figure 3). There was significant heterogeneity across these studies (*I*^2^ = 93%; *p* < 0.01) (Supplementary Figure S8). We did not find the factors such as study site, region, quality assessment, age, disease duration, number had effect on the heterogeneity of the prevalence of voiding symptoms by meta-regression.

##### Retention

A total of 14 studies investigated the prevalence of retention in 1,991 patients with PD ranging from 8 to 76% and yielding a pooled prevalence of 27% (95% CI 17–37; *I*^2^ = 96%; *p* < 0.01; Figure 2C). The pooled prevalence of PVR volume ≥ 100 ml was 4% (95% CI 1–7; *I*^2^ = 65%; *p* = 0.02; 5 studies; *n* = 439; Figure 2D).

Using the subgroup analysis, the pooled prevalence of urinary retention was 16% (95% CI 3–29; *I*^2^ = 76%; 2 studies; *n* = 240) in PD with H&Y stage <3, whereas 41% (95% CI 7–75; *I*^2^ = 97%; 2 studies; *n* = 256) in PD with H&Y stage ≥ 3. The pooled prevalence of urinary retention was 19% (95% CI 10–27; *I*^2^ = 95%; 8 studies; *n* = 1,234) in PD with age <65 years, whereas 37% (95% CI 19–56; *I*^2^ = 96%; 6 studies; *n* = 757) in PD with age ≥ 65 years (Table 2).

Using urodynamic tests, the pooled prevalence of retention was 27% (95% CI 8–46) estimated from 170 PD patients, 2 studies, with considerable heterogeneity (*I*^2^ = 88%; *p* < 0.01). Using clinical scales, the pooled prevalence of urinary incontinence was 28% (95% CI 16–40) estimated from 1,771 PD patients, 11 studies, with significant heterogeneity (*I*^2^ = 97%; *p* < 0.01); The pooled prevalence of urinary retention was 12% (95% CI 5–24; 1 study; *n* = 50) using questionnaires.

Further subgroup analysis showed that using urodynamic tests, the pooled prevalence of retention was 27% (95% CI 8–46) estimated from 170 PD patients, 2 studies, with considerable heterogeneity (*I*^2^ = 88%; *p* < 0.01). I prevalence of retention in PD was 12% (95% CI 5–24) using questionnaire and 35% (95% CI 31–41) using NMSS, respectively, all from one study. Utilizing IPSS, the estimated pooled prevalence of urinary retention was 21% (95% CI 7–35), the heterogeneity across these 5 studies in 303 PD patients was considerable (*I*^2^ = 90%; *p* < 0.01). Using SCOPA-AUT, the pooled prevalence of urinary retention was 35% (95% CI 0–75; *I*^2^ = 99%; *p* < 0.01; 3 studies; *n* = 613). Using AUA, the pooled prevalence of retention was 31% (95% CI 6–56) estimated from 477 PD patients, 2 studies, with considerable heterogeneity (*I*^2^ = 95%; *p* < 0.01) (Supplementary Figure S9; Supplementary Table 3).

Age was a source of heterogeneity related to the prevalence of retention (*p* = 0.024) by meta-regression.

##### Dysuria

The pooled prevalence of dysuria was 22% in PD (95% CI 11–34; *I*^2^ = 94%; *p* < 0.01; 4 studies; *n* = 671) (Supplementary Figure S10).

##### Hesitancy

The pooled prevalence of hesitancy was 20% in PD (95% CI 7–32; *I*^2^ = 96%; *p* < 0.01; 6 studies; *n* = 724) (Supplementary Figure S11).

##### Slow urinary stream/prolongation

The pooled prevalence of slow urinary stream was 41% in PD (95% CI 7–75; *I*^2^ = 97%; *p* < 0.01; 3 studies; *n* = 184) (Supplementary Figure S12).

##### Intermittency

The pooled prevalence of intermittency was 30% in PD (95% CI 9–51; *I*^2^ = 97%; *p* < 0.01; 8 studies; *n* = 563) (Supplementary Figure S13).

##### Spraying of urinary stream/weak stream of urine

The pooled prevalence of weak stream of urine was 22% in PD (95% CI 4–39; *I*^2^ = 95%; *p* < 0.01; 8 studies; *n* = 494) (Supplementary Figure S14).

### Sensitivity analysis

This analysis validated the result's stability.

## Discussion

This meta-analysis study indicates that LUTS and its subtypes present in a significant proportion of PD patients, occurring at rates much higher than those found in the general population. The pooled prevalence was 61% (95% CI 53–69) for LUTS, 59% (44–73) for storage symptoms, and 24% (14–33) for voiding symptoms. We found that urinary incontinence and retention have higher prevalence in PD than previously assumed. The pooled prevalence was 30% (95% CI 22–39) for urinary incontinence, 27% (17–37) for retention and 4% (1–7) for post-void residual (PVR) volume ≥100 ml, respectively. Overall, we found considerable heterogeneity among studies. The wide variety of methods were used to assess LUTS and its subtypes. The prevalence of LUTS, urinary incontinence, and urinary retention was significantly associated with diagnostic methods.

### LUTS in PD

LUTS were found in more than half of PD patients in this meta-analysis. Previous studies showed that LUTS was age-associated (Lee et al., 1998; Takahashi et al., 2022). However, it was found that patients with PD experienced significantly more LUTS than age-matched controls (10.8%) (Campos-Sousa et al., 2003). The prevalence of urinary incontinence (5.5%) in community-based elder people was lower than PD of the same age (Takahashi et al., 2022). Moreover, the prevalence of urinary retention (20.6%) in community-based men were also lower than PD in our meta-analysis (Lee et al., 1998).

However, the estimated prevalence rate of LUTS in PD was much lower than other neurodegenerative diseases, such as MSA (96%) (Sakakibara et al., 2000), progressive supranuclear palsy (PSP) (80%) (Xie et al., 2015), and dementia with Lewy bodies (91%) (Tateno et al., 2015). Previous study showed PD patients with LUTS demonstrated significantly lower dopamine transporter uptake in the striatum than patients without LUTS, according to single-photon emission computerized tomography (SPECT) imaging. LUTS were demonstrated to be correlated with putamen/caudate ratio and the degeneration of nigrostriatal dopaminergic neurons in PD patients with severe LUTS, suggesting that LUTS was associated with the degeneration of the caudate nucleus in patients with severe bladder dysfunction (Winge et al., 2005). Besides, there were potential associations between frontal executive dysfunction and LUTS or its subtypes in PD (Tkaczynska et al., 2017, 2020; Xu et al., 2019). PD dementia (PDD) and dementia with Lewy bodies were diseases with high prevalence of LUTS and characterized by cognitive impairment. Cortical alpha synuclein deposition and dysfunction of frontal cortex-basal ganglia dopaminergic circuit were hallmarks for those diseases. This further illustrated the role of frontal cortex in cognitive impairment and LUTS (Sakakibara et al., 2014). Unlike motor symptoms, LUTS were levodopa refractory symptoms, which suggest complex pathophysiological mechanisms beyond the dopaminergic system.

There were significant variations among the 27 studies evaluating LUTS prevalence in this meta-analysis. The pooled prevalence of LUTS was 59% in PD patients with mild H&Y score, whereas result for patients with severe H&Y score was 70% in this meta-analysis. This was in line with previous studies. However, the prevalence of LUTS did not differ significantly by gender, age, study site, and quality assessment of articles by subgroup analyses.

This discrepancy could be attributed to the following reasons. Firstly, the inclusion criteria of LUTS were variable among prevalence studies. LUTS can be divided into three categories including urinary storage symptoms, urinary voiding symptoms and post-voiding symptoms according to the international continence society (ICS) report (D'Ancona et al., 2019). Some studies assessing prevalence of LUTS included both storage and voiding symptoms, whereas others did not. Second, considerable heterogeneity mainly originated from the diagnostic methodological difference of LUTS. Studies assessed the prevalence of LUTS by definitions, urinary dysfunction questionnaires, clinical scales and urodynamic tests. The current meta revealed those validated ways, particularly those based on Dan-PSS and SCOPA-AUT, had higher prevalence estimates than non-validated methods, suggesting that such an approach had advantages of LUTS screening. Prevalence of LUTS assessed by IPSS scale was relatively low, suggesting that the prevalence of LUTS may be underestimated. However, some of clinical scales such as OAB-SS and AUA-SI were not validated in PD domain. OAB-SS only evaluated storage symptoms, such as urgency, frequency, nocturia and urge incontinence. NMSS only assessed urgency, frequency and nocturia, suggesting inaccuracy in diagnosing LUTS. It is quite necessary to unify the research methods. Thirdly, the heterogeneity may equally have arisen from confounders within gender, age, and disease severity. Sex and H&Y stage has been shown to be potential modifiers in our study, while age, study site and quality assessment were not.

### Storage symptoms in PD

The pooled prevalence of storage symptoms was 59%. Polyuria was the most prevalent type of LUTS in the patients with PD (65%) followed by OAB (62%), nocturia (59%), frequency (52%), urgency (46%), and incontinence (30%). The results were almost the same with those reported previously (Sakakibara et al., 2001; Tateno et al., 2021). OAB was the second most prevalent storage symptom. It was urinary urgency, usually accompanied by increased daytime frequency and/or nocturia, with urinary incontinence or without according to the International Continence Society (ICS) report (D'Ancona et al., 2019).

Storage symptoms were frequent in PD because degeneration of dopaminergic neurons might lead to urination irritative symptoms in PD (Mito et al., 2018). Striatum functionally controlled the urine storage. Disruption of D1-GABAergic direct striatal output pathway may cause bladder hyperactivity in PD which had been validated in SPECT and animal studies (Sakakibara et al., 2002; Yamamoto et al., 2005; Tateno et al., 2021). Besides, objective assessment with urodynamics showed that detrusor overactivity (DO) occur in 45–93% of PD patients, which was the most prevalent cause to OAB in PD (Sakakibara et al., 2001).

This meta-analysis revealed that the pooled prevalence of urinary incontinence was 30% in PD. Severe urinary incontinence in the first 5 year of disease is a red flag according to the MDS clinical diagnostic criteria for PD. However, a postmortem study identifying 11 cases confirmed the prevalence of urinary incontinence in PD was 82%, similar to that of MSA (87%) (Wenning et al., 1999). Although the sample size was relatively small in this study, the high prevalence rate challenged the prevailing view that urinary incontinence tended to be an exclusion criteria for PD diagnosis. However, the mean disease duration of collected cases was 16.58 years, much longer than the “5 year” indicated in the MDS diagnostic criteria. The finding suggested the possibility that disease duration might influence prevalence rate of urinary incontinence. Indeed, converging evidence pointed that urinary incontinence mainly occurs in the advanced stage of PD, ~12–15.5 years after the onset of motor symptoms (Wenning et al., 1999; Uchiyama et al., 2011; Khoo et al., 2013), because urethral sphincter function was preserved in the early stages of PD (Stocchi et al., 1997). With the increasing age and disease duration, frontal lobe damage worsened accompanied with increase of alpha synuclein deposition, leading to cognitive dysfunction and LUTS (Tkaczynska et al., 2017). Among all the studies reporting urinary incontinence, the mean disease duration ranged from 1.29 to 12.2 years. One study showed that the frequency of urinary incontinence increased in the 1st (4.1%), 2nd (12%) and 3rd (15.1%) year of follow-up with mean 2.2 years of disease duration (Stanković et al., 2019). Another study showed that the prevalence of incontinence was 8.3, 30, 43.8, and 50% in PD patients with disease duration <2 years, 2–5 years, 5–10 years, and >10 years, respectively (Liu et al., 2015). The study with the largest sample size in this meta reported that incontinence prevalence was 43% in 3,414 PD patients with mean disease duration for 9 years (Wüllner et al., 2007). All of these suggested PD patients with longer disease duration may experience more urinary incontinence. H&Y score serves as a proxy for disease severity, which was closely correlated with disease duration. The pooled prevalence of urinary incontinence was 20% in PD patients with mild H&Y score, whereas 48% in patients with severe H&Y score in this meta-analysis. The consistent results indicate the severity of the disease should also be considered as a factor affecting prevalence in addition to the course of the disease.

Although severe urinary incontinence in the first 5 years of disease is a red flag of PD diagnosis, long-standing or small amount stress incontinence in women should be excluded. Unexplained urge incontinence was a supportive diagnostic criterion of MSA according to the newest MSA diagnostic (Wenning et al., 2022). These criteria suggest simply functional incontinence alone is not enough to distinguish Parkinson's disease from multisystem atrophy. Of the 21 included studies, there were 13 studies involving urge incontinence. The pooled prevalence of urge incontinence was 32% in PD patients in this meta-analysis. Few studies reported the types of urinary incontinence in PD. Therefore, we should pay attention to the types of urinary incontinence in the analysis of urinary incontinence.

The current study also demonstrated age and gender difference in prevalence of urinary in continence. The pooled prevalence of urinary incontinence was 20% in PD patients with age <65 years, whereas 37% in PD with age ≥ 65 years in this meta-analysis. We found that the mean age was the source of heterogeneity related to the prevalence of urinary incontinence by meta-regression. The average age of 6,054 PD patients in the 21 studies varied from 57.4 to 75 years. In the study with youngest mean age, the prevalence of urinary incontinence was only 7% (Swaminath et al., 2010). These results suggested that age of PD patients contributed to the prevalence variance of urinary incontinence.

We found that the prevalence of urinary incontinence was 31% in the male PD patients, whereas 43% in female patients with PD. These results suggest that both age and gender influence the outcome of estimated prevalence.

The heterogeneity was equally possible to generate from other confounders. In the study of urinary incontinence, the diagnostic methods were not uniform, and the objective indicators are seldom applied. Using urodynamic tests, the pooled prevalence of urinary incontinence was 21%, which was less than that using scales, questionnaires, and definition. It implied that application of scales, questionnaires, and definition may have overestimated the prevalence of urinary incontinence. However, there are only two studies combining urodynamic tests. No studies in this meta-analysis reporting the urinary incontinence of pathological confirmed PD which indicated the scarcity of research in this meta. No significant publication bias of urinary incontinence was found.

The majority of studies were rated as low quality because of small sample sizes and other reason, although the results were robust in sensitivity analysis. Accounting for aforementioned factors, the prevalence results of urinary incontinence should be interpreted with caution.

### Voiding symptoms in PD

The pooled prevalence of voiding symptoms was 24%. Prolongation was the most prevalent type of voiding symptoms in PD (41%) followed by the intermittency (30%), urinary retention (27%), dysuria (22%), weak stream (22%), and hesitancy (20%). Some studies found that subclinical detrusor weakness during voiding may also occur in PD (Terayama et al., 2012; Sakakibara et al., 2016; Ogawa et al., 2017). These findings revealed that PD patients had both subclinical and clinical voiding symptoms. There was substantial heterogeneity in prevalence of voiding symptoms and its subtypes (intermittency, dysuria, weak stream, and hesitancy) among studies. The source of heterogeneity could be the limited number of articles, diverse diagnostic tools and different characteristic of participants.

Urinary retention is the most disabling voiding symptoms and often used to differentiate Parkinson's disease from multisystem atrophy. Previous studies have shown that the prevalence of urinary retention and large PVR was 43 and 14% in MSA (Ito et al., 2006; Lee et al., 2018) and PVR volume ≥ 100 ml might be an effective indicator to differentiate PD from MSA (Yamamoto et al., 2017; Lee et al., 2018; Wenning et al., 2022). Unexplained voiding difficulties with PVR volume ≥ 100 ml was one of the core clinical features to identify the clinically established MSA (Wenning et al., 2022). Furthermore, severe urinary retention in the first 5 year of disease is a red flag according to MDS clinical diagnostic criteria for PD.

Our study showed that the prevalence of retention was not low (27%) and the prevalence of large PVR (≥100 ml) was even higher than 4% in PD. Using urodynamic tests, the pooled prevalence of retention was 27% (95% CI 8–46%). Of the overall 14 studies involved urinary retention, the mean disease duration ranged from 1.97 to 12.2 years. One study revealed that the duration of disease in patients without residual urine was lower than PD patients with residual urine (4.6 vs. 7.1 years) (Zhang and Zhang, 2015). Another finding of our study was that the pooled prevalence of urinary retention was 16% in PD with H&Y stage <3, whereas 41% in PD with H&Y stage ≥ 3. The pooled prevalence of urinary retention was 19% in PD patients with age <65 years, whereas 37% in PD by age ≥ 65 years. Furthermore, we found that age was the source of heterogeneity related to the prevalence of urinary retention by meta-regression. Of 1,991 PD in the 14 studies, the median age of participants varied from 59.6 to 70.6 years. One study revealed that the patients without residual urine were younger than patients with residual urine (67.5 vs. 70.1 years) (Zhang and Zhang, 2015). A higher weighted prevalence was calculated in older people than younger people (76 vs. 18%) (Campos-Sousa et al., 2003; Irene, 2019), suggesting PD patients with older age might be more likely to experience urinary retention. Overall, when distinguishing PD from MSA with urinary retention symptoms, we should be more cautious and consider relevant factors such as age, disease duration, and disease severity.

Diagnostic tools influenced prevalence results. The prevalence of urinary retention diagnosed using questionnaire was lower than that using clinical scales and urodynamic tests., There was difference in the prevalence identified by different scales. The pooled prevalence of urinary retention was 21% (using IPSS), 31% (using AUA) and 35% (using SCOPA-AUT), which was similar to 27% (using urodynamic tests).

No significant publication bias of urinary retention was found. The majority of articles evaluated with low-moderate quality were included due to the small number of research, but there was no significant difference between high-moderate and low quality of articles. Therefore, urinary retention requires attention and investigation in PD patients. Further research on mechanisms of urinary retention is also necessary.

### Limitations

There were some limitations of the current study constituting an overall weaker level of evidence. First, present meta-analysis might be misleading given the variations in diagnostic tools of LUTS and its subtypes between studies. The heterogeneity of diagnostic tools limited the generalizability of the results. Second, only a small number of studies investigating urinary incontinence or retention were enrolled, which also indicated the scarcity of research on this area. Third, given the effect of geographical location, risk of bias, and population, high heterogeneity was found among the included studies. LUTS is associated with cognitive impairment. However, many articles did not distinguish subjects according to cognitive function. We could not conclude the definitive prevalence of LUTS and its subtypes in patients with PD. Fourth, the variables associate with LUTS, such as the UPDRS scores, laboratory indicators and related treatment methods were not included in most studies. Fifth, there was no studies reporting the prevalence of LUTS in pathological confirmed PD patients. Finally, the majority of studies were rated as low quality, although the results were robust in sensitivity analysis.

## Conclusion

This meta-analysis showed that urinary incontinence and retention are not uncommon in PD. The high prevalence of LUTS confirmed the importance of LUTS as a notable non-motor characteristic of PD. LUTS were found in more than one-half of PD patients. It was evident that patients with PD experience significantly more urinary symptoms than age-matched controls. Overall, when distinguishing PD from MSA with urinary incontinence or retention, we should be more cautious and consider relevant factors such as age, disease duration, cognitive function and disease severity. The heterogeneity of diagnostic tools of LUTS and its subtypes limited the generalizability of the results. Therefore, future studies are warranted to address this issue by developing standardized diagnostic tools of LUTS, and the utility of which needs to be validated in larger prospective trials.

## Data availability statement

The original contributions presented in the study are included in the article/Supplementary material, further inquiries can be directed to the corresponding author/s.

## Author contributions

TF conceived and designed the study. F-FL performed the meta analysis and wrote the manuscript. F-FL and Y-SC prepared the draft and figures. All authors have read, revised, and agreed to the published version of the manuscript.

## Funding

This work was supported by the National Natural Science Foundation of China (Grant Number: 82071422, 81771367, 81901151, 82020108012) and Beijing Municipal Natural Science Foundation (Grand Number: 7212031).

## Conflict of interest

The authors declare that the research was conducted in the absence of any commercial or financial relationships that could be construed as a potential conflict of interest.

## Publisher's note

All claims expressed in this article are solely those of the authors and do not necessarily represent those of their affiliated organizations, or those of the publisher, the editors and the reviewers. Any product that may be evaluated in this article, or claim that may be made by its manufacturer, is not guaranteed or endorsed by the publisher.
