# Supplementary material for: Prevalence of lower urinary tract symptoms, urinary incontinence and retention in Parkinson's disease: A systematic review and meta-analysis
Source: Front Aging Neurosci. 2022 Sep 12;14:977572. doi: 10.3389/fnagi.2022.977572 (PMC9510898; doi:10.3389/fnagi.2022.977572)
Supplement: Supplementary file 2 [file Table_2.docx]

**Supplemental Table-S2.** Included studies of LUTS or its subtypes

| LUTS or its subtypes | Reference of pooled prevalence | Reference of subgroup analysis |
| --- | --- | --- |
| LUTS | 27 studies [15; 3; 7; 16; 64; 9; 51; 29; 56; 12; 62; 22; 23; 69; 5; 25; 32; 70; 33; 55; 19; 58; 41; 50; 66; 24; 21] | [15; 9; 51; 56; 13; 38; 23; 41; 54] |
| Storage symptoms | 9 studies [15; 3; 7; 64; 51; 48; 56; 5; 58] | - |
| Voiding symptoms | 11 studies [15; 3; 7; 64; 51; 48; 56; 23; 5; 58; 52] | - |
| OAB | 7 studies [64; 23; 5; 47; 26; 66; 52] | - |
| Pollakiuria | 2 studies [69; 17] | - |
| Urgency | 42 studies [15; 39; 7; 64; 61; 27; 34; 48; 56; 67; 10; 6; 12; 18; 49; 63; 71; 11; 23; 36; 37; 69; 72; 25; 32; 40; 1; 28; 59; 2; 17; 41; 45; 50; 66; 73; 42; 44; 54; 4; 31; 52] | [46; 39; 64; 61; 8; 56; 10; 6; 13; 38; 11; 23; 36; 1; 28; 41] |
| Frequency | 27 studies [15; 39; 7; 64; 61; 27; 51; 34; 48; 56; 67; 12; 63; 23; 36; 69; 32; 40; 70; 1; 33; 45; 50; 66; 73; 31; 52] | [15; 7; 64; 61; 27; 51; 34; 48; 56; 67; 12; 63; 36; 69; 32; 40; 70; 33; 45; 66; 73; 31; 52] |
| Nocturia | 40 studies [7; 64; 61; 27; 34; 48; 10; 6; 12; 18; 49; 60; 63; 71; 62; 36; 53; 69; 72; 5; 25; 32; 70; 1; 33; 28; 43; 59; 2; 17; 41; 50; 73; 42; 44; 54; 4; 31; 52] | [39; 64; 61; 8; 56; 10; 6; 13; 38; 23; 35; 36; 53; 69; 5; 1; 28; 41; 52] |
| Incontinence | 21 studies [15; 39; 64; 61; 65; 51; 56; 67; 57; 69; 40; 47; 1; 43; 17; 45; 50; 66; 73; 30; 52] | [61; 10; 6; 23; 41; 52] |
| Dysuria | 4 studies [69; 1; 73; 30] | - |
| Hesitancy | 6 studies [39; 7; 64; 51; 68; 1] | [39; 7; 64; 51; 68; 1] |
| slow urinary stream/prolongation | 3 studies [56; 67; 1] | - |
| Retention | 14 studies[7; 64; 61; 27; 34; 48; 56; 23; 1; 59; 17; 45; 50; 73] | [64; 61; 56; 38; 23; 1] |
| PVR>100 ml | 5 studies[14; 64; 23; 69; 20] | - |
| Intermittency | 8 studies [7; 27; 34; 48; 23; 68; 1; 52] | - |
| Spraying of urinary stream/weak stream | 8 studies [7; 64; 27; 34; 48; 47; 1; 50] | - |
| Straining to void | 5 studies [64; 48; 68; 1; 17] | - |

# REFERENCE

[1] Akkoç, Y., Gök, H., Karapolat, H., Ersöz, M., Sungur, U., Köklü, K., et al. (2017). Assessment of voiding dysfunction in Parkinson's disease: Reliability and validity of the Turkish version of the Danish Prostate Symptom Score. Neurourology and Urodynamics 36(7), 1903-1909. doi: 10.1002/nau.23208.

[2] Aldaz, T., Nigro, P., Sánchez-Gómez, A., Painous, C., Planellas, L., Santacruz, P., et al. (2019). Non-motor symptoms in Huntington's disease: a comparative study with Parkinson's disease. J Neurol 266(6), 1340-1350. doi: 10.1007/s00415-019-09263-7.

[3] Araki, I., and Kuno, S. (2000). Assessment of voiding dysfunction in Parkinson's disease by the international prostate symptom score. Journal of Neurology Neurosurgery and Psychiatry 68(4), 429-433. doi: 10.1136/jnnp.68.4.429.

[4] Ayele, B. A., Zewde, Y. Z., Tafesse, A., Sultan, A., Friedman, J. H., and Bower, J. H. (2021). Non-Motor Symptoms and Associated Factors in Parkinson's Disease Patients in Addis Ababa, Ethiopia: A Multicenter Cross-Sectional Study. Ethiopian Journal of Health Sciences 31(4), 837-846. doi: 10.4314/ejhs.v31i4.19.

[5] Benli, E., Özer, F. F., Kaya, Y., Özcan, T. Ş., and Ayyildiz, A. (2016). Is there a difference between parkinson disease patients and a control group in terms of urinary symptoms and quality of life? Turkish Journal of Medical Sciences 46(6), 1665-1671. doi: 10.3906/sag-1507-148.

[6] Bostantjopoulou, S., Katsarou, Z., Karakasis, C., Peitsidou, E., Milioni, D., and Rossopoulos, N. (2013). Evaluation of non-motor symptoms in Parkinson's Disease: An underestimated necessity. Hippokratia 17(3), 214-219.

[7] Campos-Sousa, R. N., Quagliato, E., da Silva, B. B., de Carvalho, R. M., Jr., Ribeiro, S. C., and de Carvalho, D. F. (2003). Urinary symptoms in Parkinson's disease: prevalence and associated factors. Arq Neuropsiquiatr 61(2b), 359-363. doi: 10.1590/s0004-282x2003000300007.

[8] Cheon, S. M., Ha, M. S., Park, M. J., and Kim, J. W. (2008). Nonmotor symptoms of Parkinson's disease: Prevalence and awareness of patients and families. Parkinsonism and Related Disorders 14(4), 286-290. doi: 10.1016/j.parkreldis.2007.09.002.

[9] Coelho, M., Marti, M. J., Tolosa, E., Ferreira, J. J., Valldeoriola, F., Rosa, M., et al. (2010). Late-stage Parkinson's disease: the Barcelona and Lisbon cohort. J Neurol 257(9), 1524-1532. doi: 10.1007/s00415-010-5566-8.

[10] Crosiers, D., Pickut, B., Theuns, J., De Deyn, P. P., Van Broeckhoven, C., Martinez-Martin, P., et al. (2012). Non-motor symptoms in a Flanders-Belgian population of 215 Parkinson's disease patients as assessed by the Non-Motor Symptoms Questionnaire. American Journal of Neurodegenerative Diseases 1(2), 160-167.

[11] de Souza, A., Pai Kakode, V. R., D'Costa, Z., and Bhonsle, S. K. (2015). Non-motor symptoms in Indian patients with Parkinson's disease. Basal Ganglia 5(4), 89-93. doi: 10.1016/j.baga.2015.09.002.

[12] Guo, X., Song, W., Chen, K., Chen, X., Zheng, Z., Cao, B., et al. (2013). Disease duration-related differences in non-motor symptoms: A study of 616 Chinese Parkinson's disease patients. Journal of the Neurological Sciences 330(1-2), 32-37. doi: 10.1016/j.jns.2013.03.022.

[13] Guo, X., Song, W., Chen, K., Chen, X., Zheng, Z., Cao, B., et al. (2013). Gender and onset age-related features of non-motor symptoms of patients with Parkinson's disease - A study from Southwest China. Parkinsonism and Related Disorders 19(11), 961-965. doi: 10.1016/j.parkreldis.2013.06.009.

[14] Hahn, K., and Ebersbach, G. (2005). Sonographic assessment of urinary retention in multiple system atrophy and idiopathic Parkinson's disease. Movement Disorders 20(11), 1499-1502. doi: 10.1002/mds.20586.

[15] Hattori, T., Yasuda, K., Kita, K., and Hirayama, K. (1992). Voiding Dysfunction in Parkinsons-Disease. Japanese Journal of Psychiatry and Neurology 46(1), 181-186.

[16] Hobson, P., Islam, W., Roberts, S., Adhiyman, V., and Meara, J. (2003). The risk of bladder and autonomic dysfunction in a community cohort of Parkinson's disease patients and normal controls. Parkinsonism & Related Disorders 10(2), 67-71. doi: 10.1016/j.parkreldis.2003.07.001.

[17] Irene, R. (2019). Genitourinary Dysfunction Prevalence in Parkinson Disease Patients. ARS Medica Tomitana 25(1), 6-10. doi: 10.2478/arsm-2019-0002.

[18] Khoo, T. K., Yarnall, A. J., Duncan, G. W., Coleman, S., O'Brien, J. T., Brooks, D. J., et al. (2013). The spectrum of nonmotor symptoms in early Parkinson disease. Neurology 80(3), 276-281. doi: 10.1212/WNL.0b013e31827deb74.

[19] Kim, K. J., Jeong, S. J., and Kim, J. M. (2018). Neurogenic bladder in progressive supranuclear palsy: A comparison with Parkinson's disease and multiple system atrophy. Neurourol Urodyn 37(5), 1724-1730. doi: 10.1002/nau.23496.

[20] Lee, Y. H., Lee, J. E., Ryu, D. W., Oh, Y. S., Lee, K. S., Hong, S. H., et al. (2018). Urinary dysfunctions and post-void residual urine in typical and atypical Parkinson diseases. Journal of Parkinson's Disease 8(1), 145-152. doi: 10.3233/JPD-171254.

[21] Lichter, D. G., Benedict, R. H. B., and Hershey, L. A. (2021). Freezing of Gait in Parkinson's Disease: Risk Factors, Their Interactions, and Associated Nonmotor Symptoms. Parkinsons Dis 2021, 8857204. doi: 10.1155/2021/8857204.

[22] Liu, W. M., Lin, R. J., Yu, R. L., Tai, C. H., Lin, C. H., and Wu, R. M. (2015). The impact of nonmotor symptoms on quality of life in patients with parkinson’s disease in Taiwan. Neuropsychiatric Disease and Treatment 11, 2865-2873. doi: 10.2147/NDT.S88968.

[23] Liu, Z., Uchiyama, T., Sakakibara, R., and Yamamoto, T. (2015). Underactive and overactive bladders are related to motor function and quality of life in Parkinson's disease. International Urology and Nephrology 47(5), 751-757. doi: 10.1007/s11255-015-0951-y.

[24] Martinez-Ramirez, D., Velazquez-Avila, E. S., Almaraz-Espinoza, A., Gonzalez-Cantú, A., Vazquez-Elizondo, G., Overa-Posada, D., et al. (2020). Lower urinary tract and gastrointestinal dysfunction are common in early parkinson's disease. Parkinson's Disease 2020, 1694547. doi: 10.1155/2020/1694547.

[25] Mekawichai, P., Kunadisorn, S., Tungkasereerak, C., and Saetang, S. (2016). Non-motor symptoms in thai Parkinson’s disease patients and the correlation with motor symptoms. Neurology Asia 21(1), 41-46.

[26] Mito, Y., Yabe, I., Yaguchi, H., Takei, T., Terae, S., and Tajima, Y. (2018). Relation of overactive bladder with motor symptoms and dopamine transporter imaging in drug-naive Parkinson's disease. Parkinsonism & Related Disorders 50, 37-41. doi: 10.1016/j.parkreldis.2018.02.017.

[27] Mohammed, E. S., and Ragab, M. M. (2010). Idiopathic Parkinson's disease: Lower urinary tract dysfunctions and urodynamic abnormalities. Egyptian Journal of Neurology, Psychiatry and Neurosurgery 47(3), 381-386.

[28] Mukhtar, S., Imran, R., Zaheer, M., and Tariq, H. (2018). Frequency of non-motor symptoms in Parkinson's disease presenting to tertiary care centre in Pakistan: an observational, cross-sectional study. BMJ Open 8(5), e019172. doi: 10.1136/bmjopen-2017-019172.

[29] Muller, B., Larsen, J. P., Wentzel-Larsen, T., Skeie, G. O., and Tysnes, O. B. (2011). Autonomic and Sensory Symptoms and Signs in Incident, Untreated Parkinson's Disease: Frequent but Mild. Movement Disorders 26(1), 65-72. doi: 10.1002/mds.23387.

[30] Nakahara, K., Kurisaki, R., Nakane, S., Sakamoto, T., Ikeda, T., Yamashita, T., et al. (2020). Correlation between urinary incontinence and psychosis in patients with advanced-stage Parkinson’s disease. Neurology and Clinical Neuroscience 8(6), 385-389. doi: 10.1111/ncn3.12435.

[31] Ojo, O. O., Wahab, K. W., Bello, A. H., Abubakar, S. A., Ekeh, B. C., Otubogun, F. M., et al. (2021). A Cross-Sectional Comprehensive Assessment of the Profile and Burden of Non-motor Symptoms in Relation to Motor Phenotype in the Nigeria Parkinson Disease Registry Cohort. Movement Disorders Clinical Practice 8(8), 1206-1215. doi: 10.1002/mdc3.13346.

[32] Ou, R., Yang, J., Cao, B., Wei, Q., Chen, K., Chen, X., et al. (2016). Progression of non-motor symptoms in Parkinson's disease among different age populations: A two-year follow-up study. Journal of the Neurological Sciences 360, 72-77. doi: 10.1016/j.jns.2015.11.047.

[33] Radicati, F. G., Martinez Martin, P., Fossati, C., Chaudhuri, K. R., Torti, M., Rodriguez Blazquez, C., et al. (2017). Non motor symptoms in progressive supranuclear palsy: prevalence and severity. NPJ Parkinsons Dis 3, 35. doi: 10.1038/s41531-017-0037-x.

[34] Ragab, M. M., and Mohammed, E. S. (2011). Idiopathic Parkinson's disease patients at the urologic clinic. Neurourology and Urodynamics 30(7), 1258-1261. doi: 10.1002/nau.20983.

[35] Rana, A. Q., Paul, D. A., Qureshi, A. M., Ghazi, A., Alenezi, S., Rana, M. A., et al. (2015). Association between nocturia and anxiety in Parkinson's disease. Neurol Res 37(7), 563-567. doi: 10.1179/1743132815y.0000000010.

[36] Ravan, A., Ahmad, F. M. H., Chabria, S., Gadhari, M., and Sankhla, C. S. (2015). Non-motor symptoms in an Indian cohort of Parkinson′s disease patients and correlation of progression of non-motor symptoms with motor worsening. Neurology India 63(2), 166-174. doi: 10.4103/0028-3886.156276.

[37] Raven, A., Ahmad, F. M. H., Chabria, S., Gadhari, M., and Sankhla, C. S. (2015). Non-motor symptoms in an Indian cohort of Parkinson's disease patients and correlation of progression of non-motor symptoms with motor worsening. Neurology India 63(2), 166-174. doi: 10.4103/0028-3886.156276.

[38] Robinson, J. P., Bradway, C. W., Bunting-Perry, L., Avi-Itzhak, T., Mangino, M., Chittams, J., et al. (2013). Lower urinary tract symptoms in men with Parkinson disease. J Neurosci Nurs 45(6), 382-392; quiz E381-382. doi: 10.1097/JNN.0b013e3182a3cf67.

[39] Sakakibara, R., Shinotoh, H., Uchiyama, T., Sakuma, M., Kashiwado, M., Yoshiyama, M., et al. (2001). Questionnaire-based assessment of pelvic organ dysfunction in Parkinson's disease. Autonomic Neuroscience: Basic and Clinical 92(1-2), 76-85. doi: 10.1016/S1566-0702(01)00295-8.

[40] Sakushima, K., Yamazaki, S., Fukuma, S., Hayashino, Y., Yabe, I., Fukuhara, S., et al. (2016). Influence of urinary urgency and other urinary disturbances on falls in Parkinson's disease. J Neurol Sci 360, 153-157. doi: 10.1016/j.jns.2015.11.055.

[41] Sanchez-Martinez, C. M., Choreno-Parra, J. A., Placencia-Alvarez, N., Nunez-Orozco, L., and Guadarrama-Ortiz, P. (2019). Frequency and Dynamics of Non-motor Symptoms Presentation in Hispanic Patients With Parkinson Disease. Frontiers in Neurology 10, 1197. doi: 10.3389/fneur.2019.01197.

[42] Schrag, A., Hommel, A. L. A. J., Lorenzl, S., Meissner, W. G., Odin, P., Coelho, M., et al. (2020). The late stage of Parkinson's –results of a large multinational study on motor and non-motor complications. Parkinsonism and Related Disorders 75, 91-96. doi: 10.1016/j.parkreldis.2020.05.016.

[43] Serra, M. C., Landry, A., Juncos, J. L., Markland, A. D., Burgio, K. L., Goode, P. S., et al. (2018). Increased odds of bladder and bowel symptoms in early Parkinson's disease. Neurourology and Urodynamics 37(4), 1344-1348. doi: 10.1002/nau.23443.

[44] Shahid, M. A., Khan, A. M., Iqbal, W., and Ahmad, S. (2020). The frequency of non motor symptoms (NMS) among patients with Parkinson's disease in a tertiary care hospital. Pakistan Journal of Medical and Health Sciences 14(1), 315-316.

[45] Shin, J. H., Park, K. W., Heo, K. O., Chung, S. J., and Choo, M. S. (2019). Urodynamic study for distinguishing multiple system atrophy from Parkinson disease. Neurology 93(10), e946-e953. doi: 10.1212/wnl.0000000000008053.

[46] Singer, C., Weiner, W. J., and Sanchez-Ramos, J. R. (1992). Autonomic dysfunction in men with Parkinson's disease. European Neurology 32(3), 134-140. doi: 10.1159/000116810.

[47] Smith, M., Seth, J., Batla, A., Hofereiter, J., Bhatia, K. P., and Panicker, J. N. (2016). Nocturia in Patients With Parkinson's Disease. Movement Disorders Clinical Practice 3(2), 168-172. doi: 10.1002/mdc3.12279.

[48] Soliman, R. H., and Masoud, A. (2011). Voiding dysfunction in Parkinson's disease. Egyptian Journal of Neurology, Psychiatry and Neurosurgery 48(2), 117-122.

[49] Špica, V., Pekmezović, T., Svetel, M., and Kostić, V. S. (2013). Prevalence of non-motor symptoms in young-onset versus late-onset Parkinson's disease. Journal of Neurology 260(1), 131-137. doi: 10.1007/s00415-012-6600-9.

[50] Stanković, I., Petrović, I., Pekmezović, T., Marković, V., Stojković, T., Dragašević-Mišković, N., et al. (2019). Longitudinal assessment of autonomic dysfunction in early Parkinson's disease. Parkinsonism and Related Disorders 66, 74-79. doi: 10.1016/j.parkreldis.2019.07.008.

[51] Swaminath, P. V., Ragothaman, M., Koshy, S., Sarangmath, N., Adhyam, M., Subbakrishna, D. K., et al. (2010). Urogenital symptoms in Parkinson's disease and multiple system atrophy-Parkinsonism: at onset and later. J Assoc Physicians India 58, 86-90.

[52] Tateno, F., Sakakibara, R., Ogata, T., Aiba, Y., Takahashi, O., and Sugiyama, M. (2021). The relationship between lower urinary tract function and (123)ioflupane scintigraphy in drug-naive Parkinson's disease. Autonomic Neuroscience-Basic & Clinical 233, 102813. doi: 10.1016/j.autneu.2021.102813.

[53] Telarovic, S., Mijatovic, D., and Telarovic, I. (2015). Effects of various factors on sleep disorders and quality of life in Parkinson's disease. Acta Neurologica Belgica 115(4), 615-621. doi: 10.1007/s13760-015-0478-0.

[54] Tkaczynska, Z., Becker, S., Maetzler, W., Timmers, M., Van Nueten, L., Sulzer, P., et al. (2020). Executive Function Is Related to the Urinary Urgency in Non-demented Patients With Parkinson’s Disease. Frontiers in Aging Neuroscience 12, 55. doi: 10.3389/fnagi.2020.00055.

[55] Tkaczynska, Z., Pilotto, A., Becker, S., Graber-Sultan, S., Berg, D., and Liepelt-Scarfone, I. (2017). Association between cognitive impairment and urinary dysfunction in Parkinson's disease. Journal of Neural Transmission 124(5), 543-550. doi: 10.1007/s00702-017-1690-2.

[56] Uchiyama, T., Sakakibara, R., Yamamoto, T., Ito, T., Yamaguchi, C., Awa, Y., et al. (2011). Urinary dysfunction in early and untreated Parkinson's disease. Journal of Neurology, Neurosurgery and Psychiatry 82(12), 1382-1386. doi: 10.1136/jnnp.2011.241075.

[57] Vale, T. C., Caramelli, P., and Cardoso, F. (2015). Clinicoradiological comparison between vascular parkinsonism and Parkinson's disease. Journal of Neurology, Neurosurgery and Psychiatry 86(5), 547-553. doi: 10.1136/jnnp-2014-307867.

[58] Valentino, F., Bartolotta, T. V., Cosentino, G., Mastrilli, S., Arnao, V., Aridon, P., et al. (2018). Urological dysfunctions in patients with Parkinson's disease: Clues from clinical and non-invasive urological assessment. BMC Neurology 18(1), 148. doi: 10.1186/s12883-018-1151-z.

[59] Valldeoriola, F., Salvador, A., Gómez-Arguelles, J. M., Marey, J., Moya, M., Ayuga, Á., et al. (2018). The effects of transdermal rotigotine on non-motor symptoms of Parkinson's disease: a multicentre, observational, retrospective, post-marketing study. Int J Neurosci 128(4), 369-375. doi: 10.1080/00207454.2017.1387111.

[60] Vaughan, C. P., Juncos, J. L., Trotti, L. M., Johnson Ii, T. M., and Bliwise, D. L. (2013). Nocturia and overnight polysomnography in Parkinson disease. Neurourology and Urodynamics 32(8), 1080-1085. doi: 10.1002/nau.22365.

[61] Verbaan, D., Marinus, J., Visser, M., van Rooden, S. M., Stiggelbout, A. M., and van Hilten, J. J. (2007). Patient-reported autonomic symptoms in Parkinson disease. Neurology 69(4), 333-341. doi: 10.1212/01.wnl.0000266593.50534.e8.

[62] Vongvaivanich, K., Nidhinandana, S., Udommongkol, C., Chairungsaris, P., Chinvarun, Y., Wongmek, W., et al. (2014). Non-motor symptoms in Thai patients with Parkinson’s disease Studied at Phramongkutklao Hospital. Journal of the Medical Association of Thailand 97, S159-S167.

[63] Weerkamp, N. J., Tissingh, G., Poels, P. J. E., Zuidema, S. U., Munneke, M., Koopmans, R., et al. (2013). Nonmotor Symptoms in Nursing Home Residents with Parkinson's Disease: Prevalence and Effect on Quality of Life. Journal of the American Geriatrics Society 61(10), 1714-1721. doi: 10.1111/jgs.12458.

[64] Winge, K., Skau, A. M., Stimpel, H., Nielsen, K. K., and Werdelin, L. (2006). Prevalence of bladder dysfunction in Parkinsons disease. Neurourology and Urodynamics 25(2), 116-122. doi: 10.1002/nau.20193.

[65] Wüllner, U., Schmitz-Hübsch, T., Antony, G., Fimmers, R., Spottke, A., Oertel, W. H., et al. (2007). Autonomic dysfunction in 3414 Parkinson's disease patients enrolled in the German Network on Parkinson's disease (KNP e.V.): The effect of ageing. European Journal of Neurology 14(12), 1405-1408. doi: 10.1111/j.1468-1331.2007.01982.x.

[66] Xu, D., Han, S., Wang, J., and Feng, J. (2019). Relationship between Lower Urinary Tract Dysfunction and Clinical Features in Chinese Parkinson's Disease Patients. Parkinsons Dis 2019, 6820937. doi: 10.1155/2019/6820937.

[67] Yamamoto, T., Sakakibara, R., Uchiyama, T., Yamaguchi, C., Nomura, F., Ito, T., et al. (2011). Pelvic organ dysfunction is more prevalent and severe in MSA-P compared to parkinson's disease. Neurourology and Urodynamics 30(1), 102-107. doi: 10.1002/nau.20948.

[68] Yamamoto, T., Tateno, F., Sakakibara, R., Furukawa, S., Asahina, M., Uchiyama, T., et al. (2016). Urinary dysfunction in progressive supranuclear palsy compared with other parkinsonian disorders. PLoS ONE 11(2), e0149278. doi: 10.1371/journal.pone.0149278.

[69] Zhang, L. M., and Zhang, X. P. (2015). Investigation of urination disorder in Parkinson’s disease. Chinese Medical Journal 128(21), 2906-2912. doi: 10.4103/0366-6999.168049.

[70] Zhang, S. S., Ou, R. W., Chen, X. P., Yang, J., Zhao, B., Yuan, X. Q., et al. (2016). Correlative factors of cognitive dysfunction in PD patients: a cross-sectional study from Southwest China. Neurological Research 38(5), 434-440. doi: 10.1080/01616412.2016.1139320.

[71] Zhou, M. Z., Gan, J., Wei, Y. R., Ren, X. Y., Chen, W., and Liu, Z. G. (2013). The association between non-motor symptoms in Parkinson's disease and age at onset. Clinical Neurology and Neurosurgery 115(10), 2103-2107. doi: 10.1016/j.clineuro.2013.07.027.

[72] Zis, P., Martinez-Martin, P., Sauerbier, A., Rizos, A., Sharma, J. C., Worth, P. F., et al. (2015). Non-motor symptoms burden in treated and untreated early Parkinson's disease patients: argument for non-motor subtypes. Eur J Neurol 22(8), 1145-1150. doi: 10.1111/ene.12733.

[73] Zong, H., Meng, F., Zhang, Y., Wei, G., and Zhao, H. (2019). Clinical study of the effects of deep brain stimulation on urinary dysfunctions in patients with Parkinson’s disease. Clinical Interventions in Aging 14, 1159-1166. doi: 10.2147/CIA.S204368.
