# Supplementary material for: Prevalence of lower urinary tract symptoms, urinary incontinence and retention in Parkinson's disease: A systematic review and meta-analysis
Source: Front Aging Neurosci. 2022 Sep 12;14:977572. doi: 10.3389/fnagi.2022.977572 (PMC9510898; doi:10.3389/fnagi.2022.977572)
Supplement: Supplementary file 3 [file Table_3.docx]

**Supplementary Table S3.** Subgroup analysis of LUTS and retention prevalence based on different diagnostic methods

|  | Number of studies | Number of PD patients | Prevalence  Range | Prevalence mean(%), 95% CI | I^2^ |
| --- | --- | --- | --- | --- | --- |
| IPSS | 3 | 1331 | 24 to 69 | 39(11 to 87) | 94 |
| SCOPA | 3 | 569 | 49 to 95 | 79(50 to 100) | 98 |
| AUA | 1 | 61 | - | 39(27 to 53) | - |
| NMSS | 10 | 2172 | 26 to 89 | 60(48 to 72) | 98 |
| Questionnaire | 5 | 638 | 46 to 84 | 60(48 to 71) | 90 |
| Definition | 2 | 201 | 55 to 60 | 58(51 to 65) | 0 |
| Dan-PSS | 1 | 107 | - | 88(80 to 93) | - |
| OABSS | 1 | 100 | - | 89(81 to 94) | - |

|  | Number of studies | Number of PD patients | Prevalence  Range | Prevalence mean(%), 95% CI | I^2^ |
| --- | --- | --- | --- | --- | --- |
| IPSS | 5 | 303 | 8 to 48 | 21(7 to 35) | 90 |
| SCOPA | 3 | 613 | 12 to 79 | 35(0 to 75) | 99 |
| AUA | 2 | 477 | 18 to 44 | 31(6 to 56) | 95 |
| NMSS | 1 | 378 | - | 35(31to 41) | - |
| Questionnaire | 1 | 50 | - | 12(5 to 24) | - |
| Urodynamic tests | 2 | 170 | 17 to 37 | 27(8 to 46) | 88 |
