# Supplementary material for: Prevalence of lower urinary tract symptoms, urinary incontinence and retention in Parkinson's disease: A systematic review and meta-analysis
Source: Front Aging Neurosci. 2022 Sep 12;14:977572. doi: 10.3389/fnagi.2022.977572 (PMC9510898; doi:10.3389/fnagi.2022.977572)

**Supplementary Figure-S1**. Subgroup analysis of LUTS prevalence based on different diagnostic methods


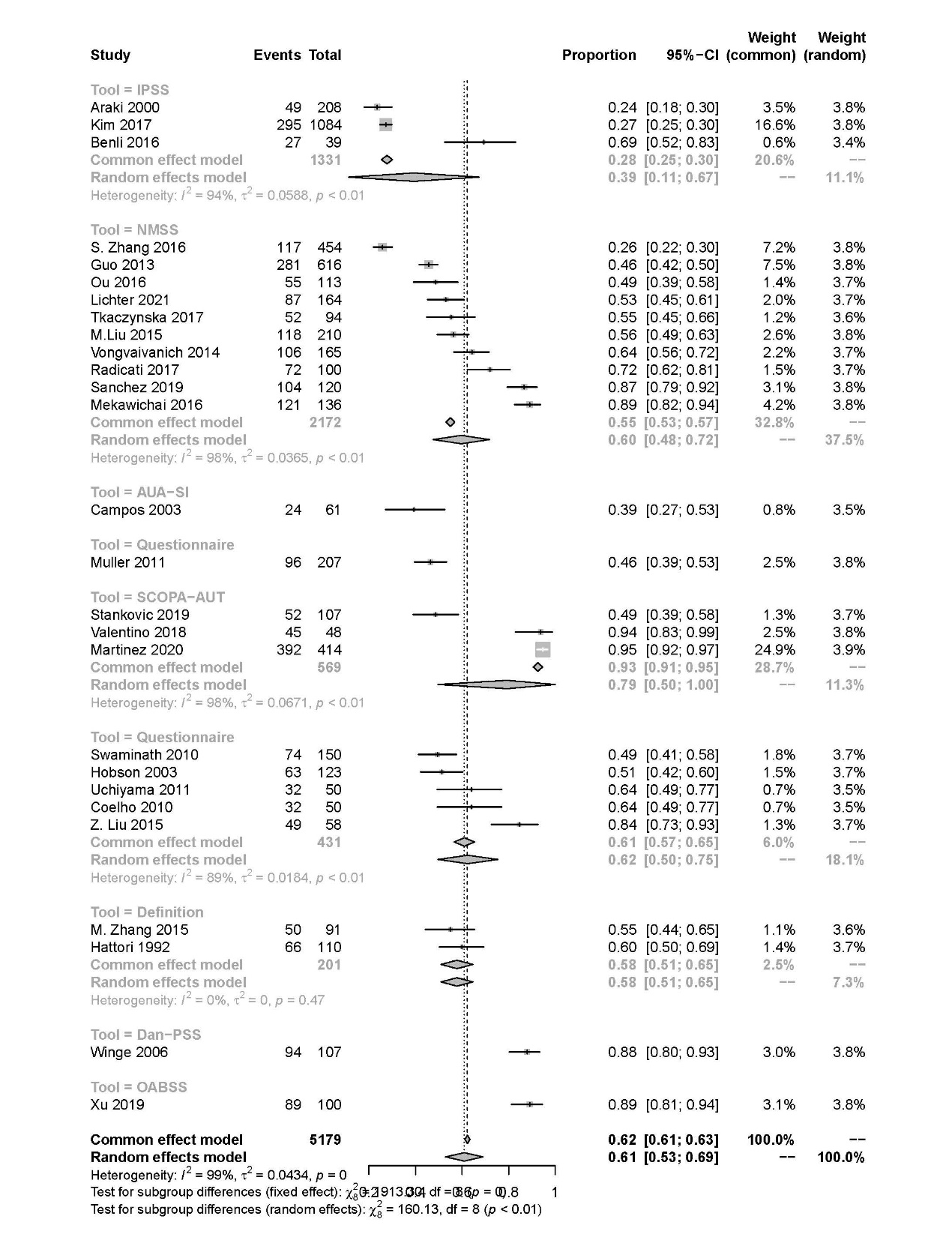


**Supplementary Figure-S2**. Forest plot showing the prevalence of storage symptoms in PD


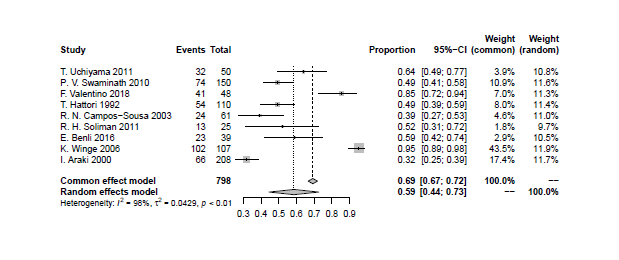


**Supplementary Figure-S3**. Forest plot showing the prevalence of OAB in PD


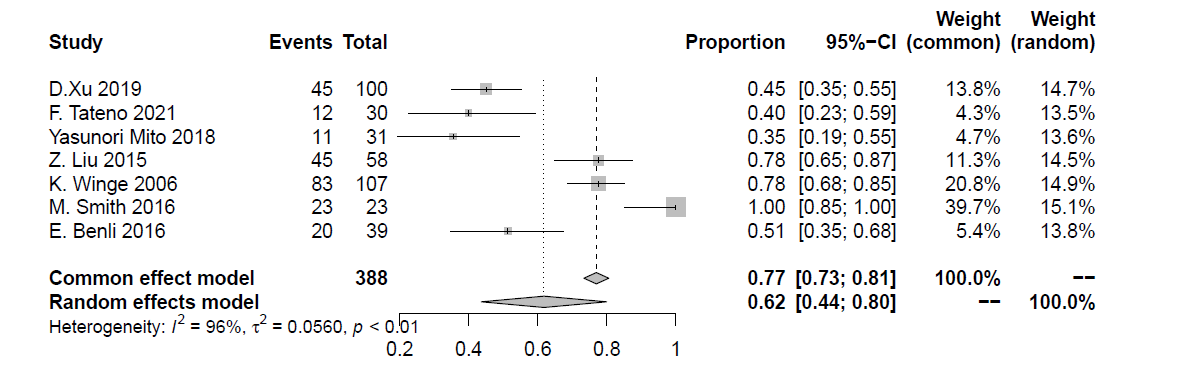


**Supplementary Figure-S4**. Forest plot showing the prevalence of urgency in PD


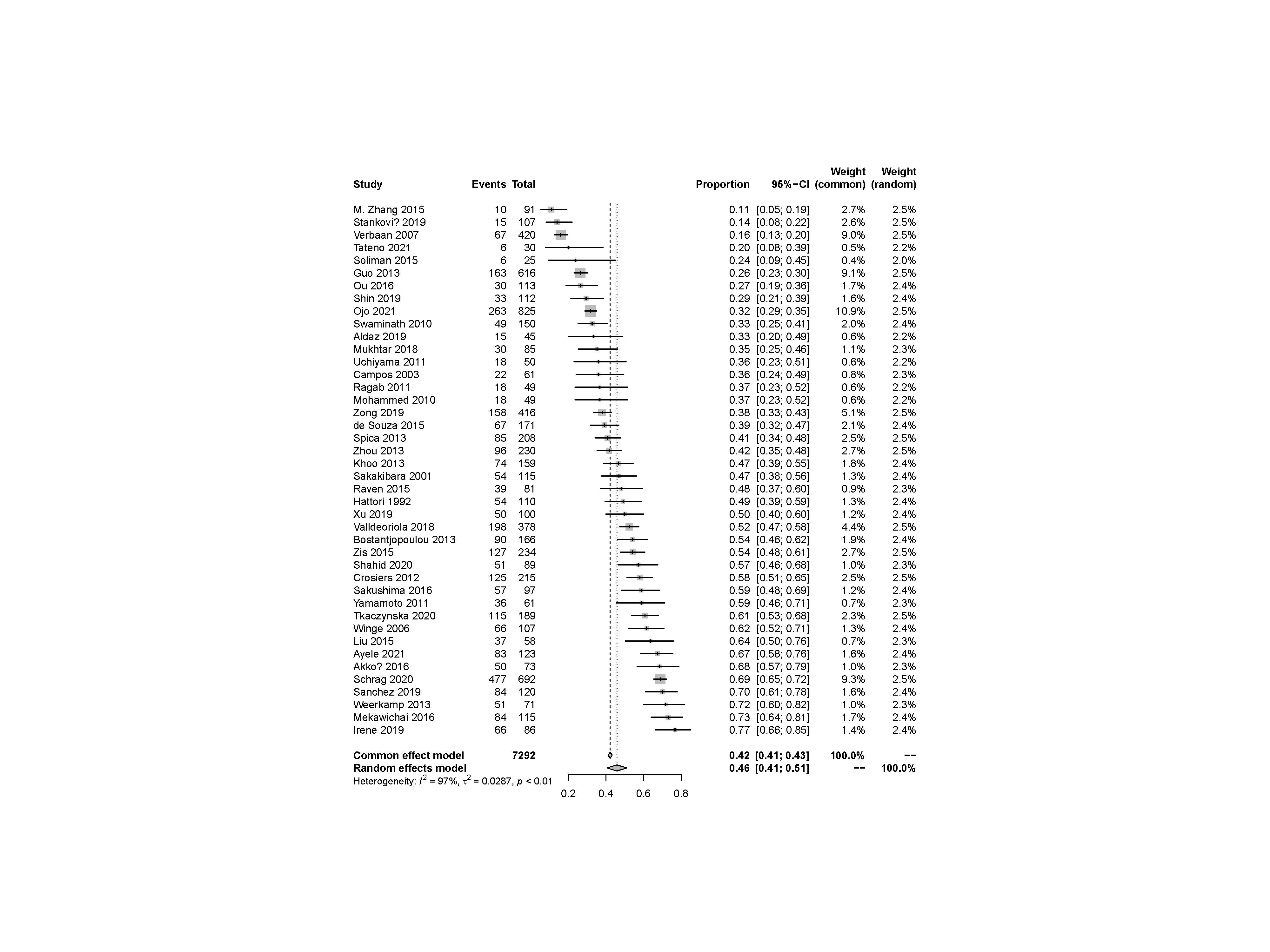


**Supplementary Figure-S5**. Forest plot showing the prevalence of urinary frequency in PD


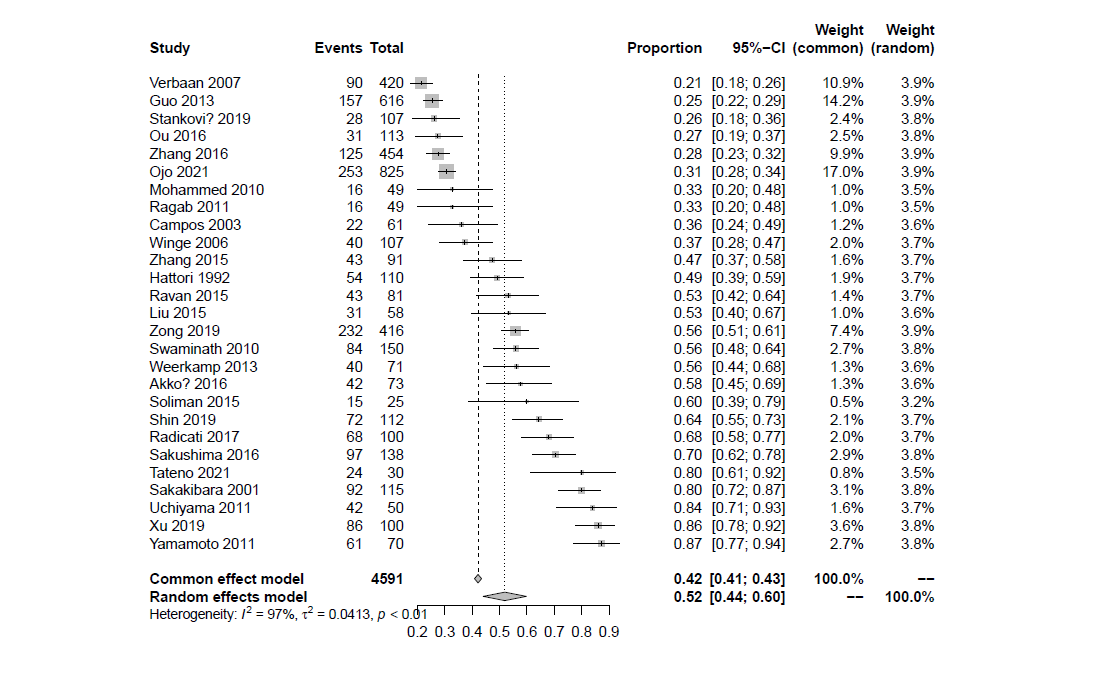


**Supplementary Figure-S6**. Forest plot showing the prevalence of nocturia in PD


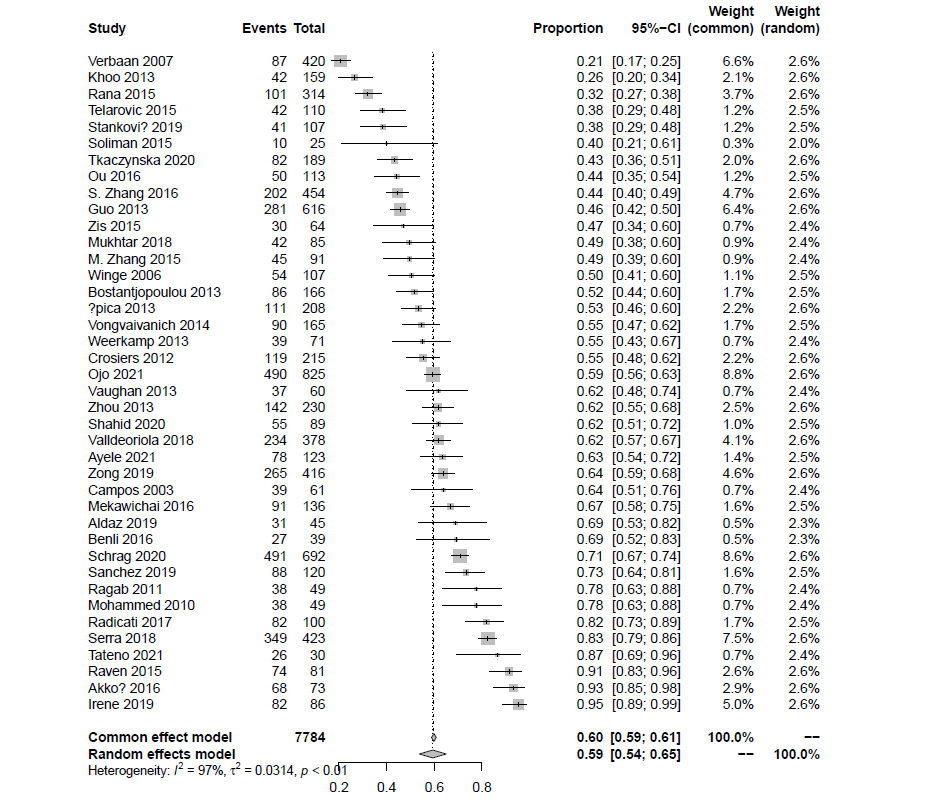


**Supplementary Figure-S7**. Forest plot showing the prevalence of pollakiuria in PD


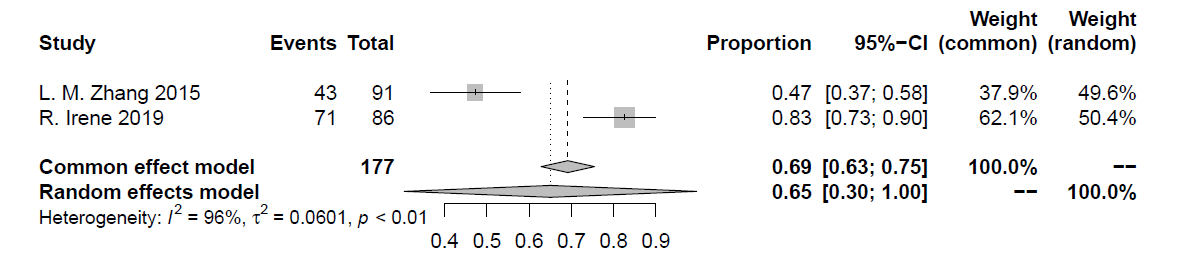


**Supplementary Figure-S8**. Forest plot showing the prevalence of voiding symptoms in PD


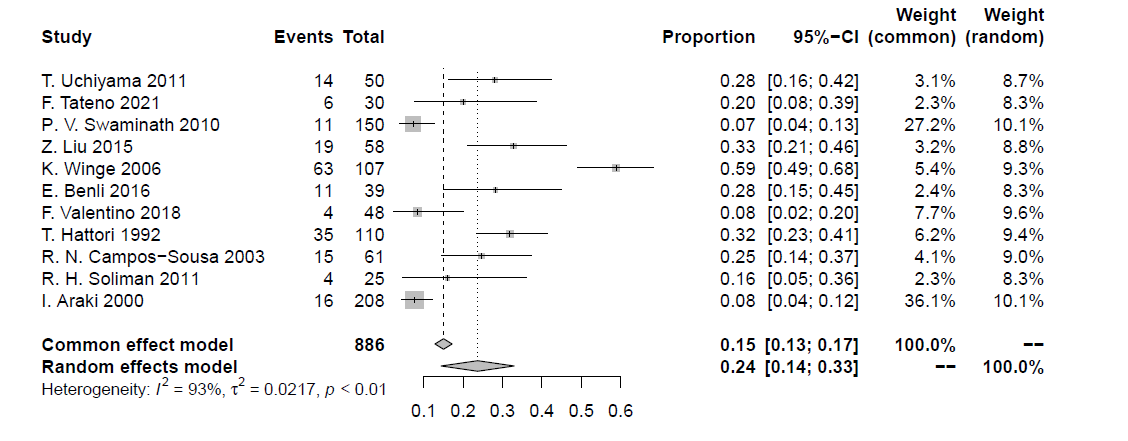


**Supplementary Figure-S9**. Subgroup analysis of retention prevalence based on different diagnostic methods


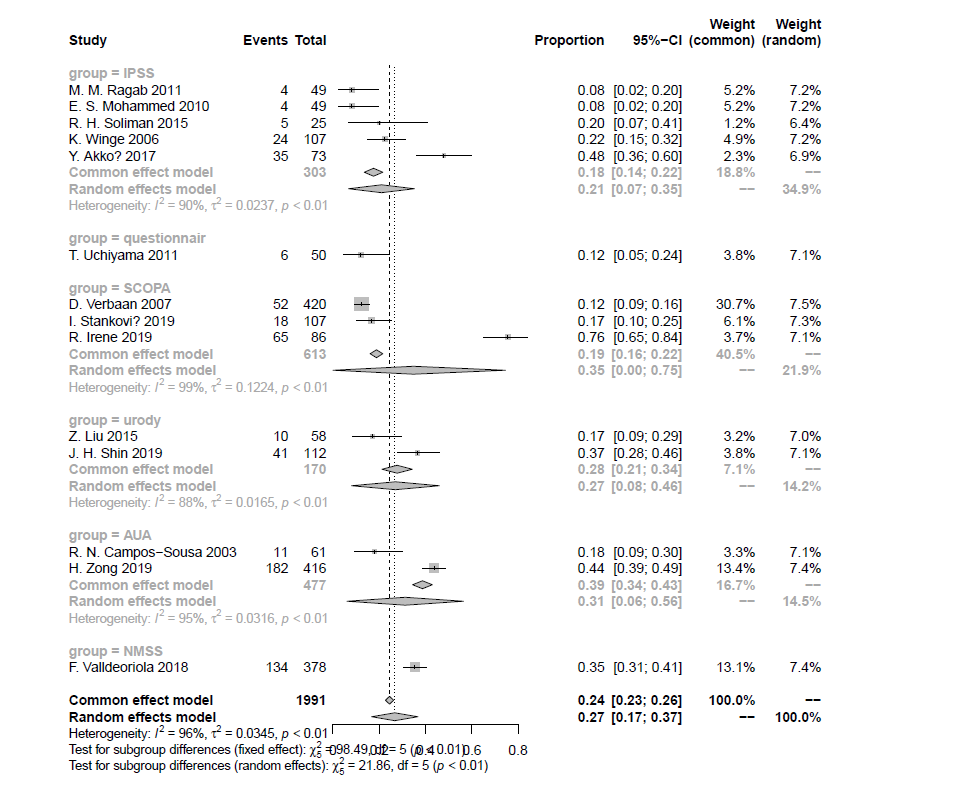


**Supplementary Figure-S10**. Forest plot showing the prevalence of dysuria in PD


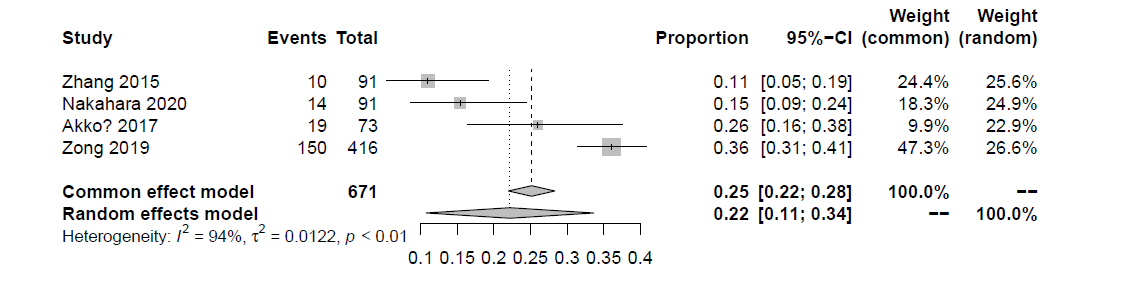


**Supplementary Figure-S11**. Forest plot showing the prevalence of hesitancy in PD


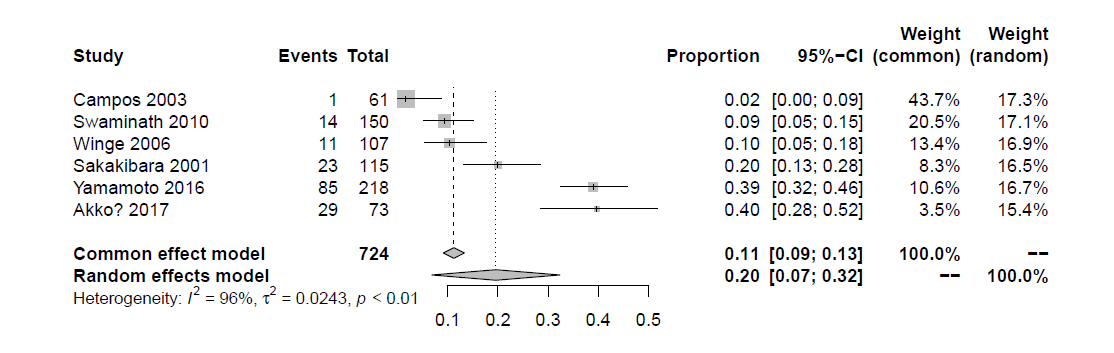


**Supplementary Figure-S12**. Forest plot showing the prevalence of prolongation in PD


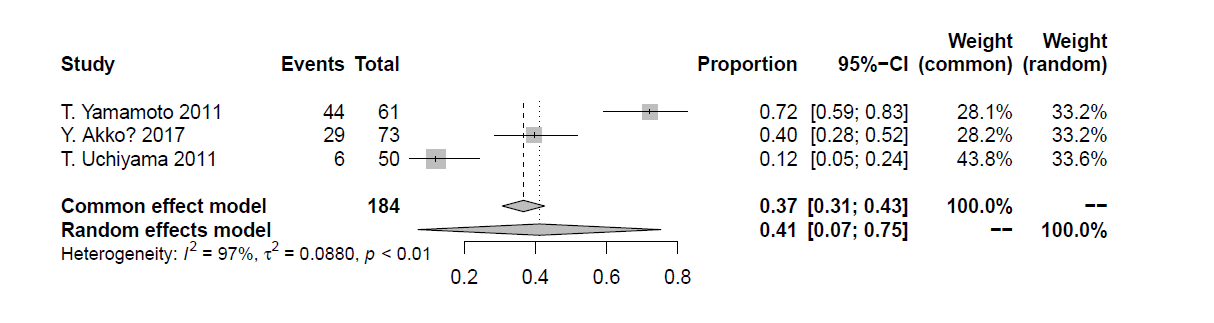


**Supplementary Figure-S13**. Forest plot showing the prevalence of intermittency in PD


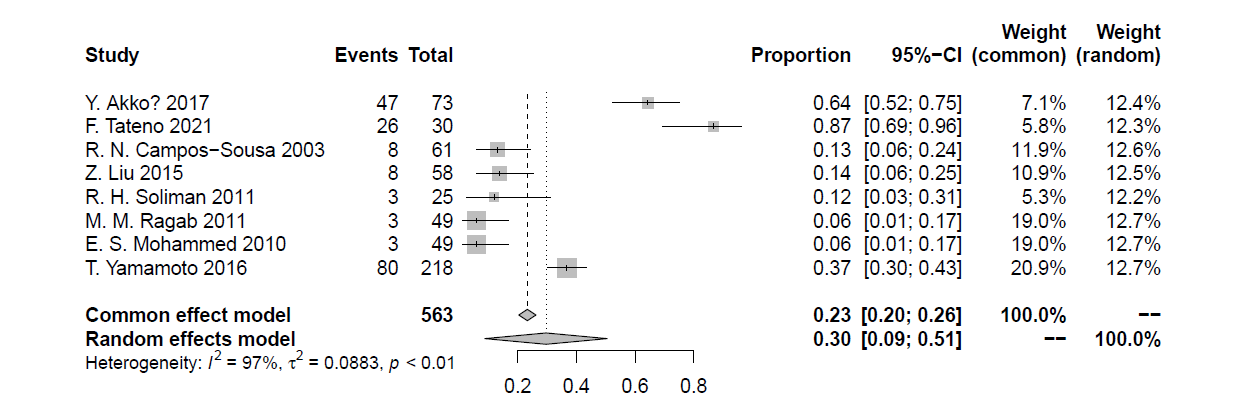


**Supplementary Figure-S14**. Forest plot showing the prevalence of weak stream of urice in PD


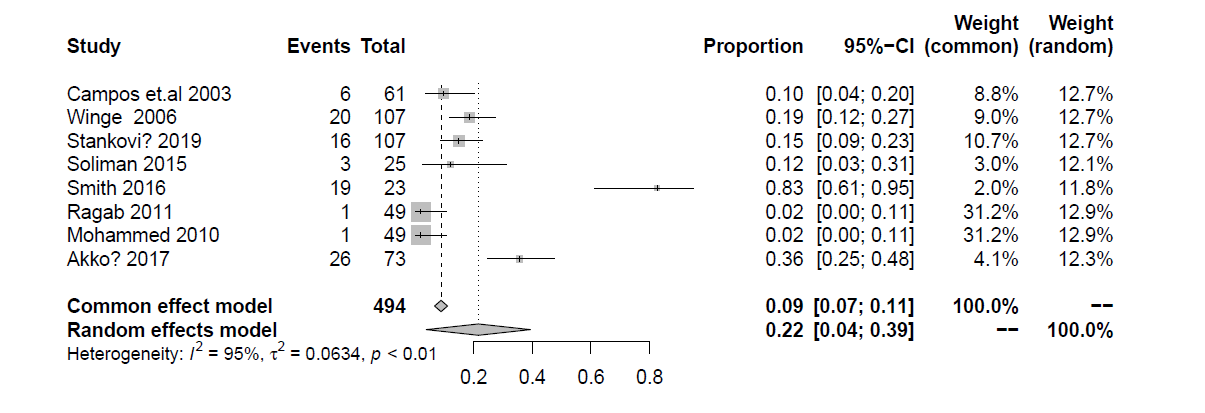

Supplement: Supplementary file 4 [file Data_Sheet_1.docx]
